# Frequency regulation in a hybrid renewable power grid: an effective strategy utilizing load frequency control and redox flow batteries

**DOI:** 10.1038/s41598-024-58189-2

**Published:** 2024-04-26

**Authors:** Ahmed H. A. Elkasem, Salah Kamel, Mohamed Khamies, Loai Nasrat

**Affiliations:** 1 Department of Electrical Engineering, Faculty of Engineering, Aswan University, Aswan, 81542 Egypt; 2 Department of Electrical Engineering, Faculty of Engineering, Sohag University, Sohag, 82524 Egypt

**Keywords:** Fuzzy-PID + (T ${I}^{\lambda }{D}^{\mu }$) controller, Crayfish optimization algorithm, Load frequency control, Renewable energy resources, Controlled redox flow batteries, Communication delay time, Electrical and electronic engineering, Energy infrastructure

## Abstract

Renewable energy sources (RESs) have become integral components of power grids, yet their integration presents challenges such as system inertia losses and mismatches between load demand and generation capacity. These issues jeopardize grid stability. To address this, an effective approach is proposed, combining enhanced load frequency control (LFC) (i.e., fuzzy PID- T ${I}^{\lambda }{D}^{\mu }$) with controlled energy storage systems, specifically controlled redox flow batteries (CRFBs), to mitigate uncertainties arising from RES integration. The optimization of this strategy's parameters is achieved using the crayfish optimization algorithm (COA), known for its global optimization capabilities and balance between exploration and exploitation. Performance evaluation against conventional controllers (PID, FO-PID, FO-(PD-PI)) confirms the superiority of the proposed approach in LFC. Extensive testing under various load disturbances, high renewables penetration, and communication delays ensures its effectiveness in minimizing disruptions. Validation using a standardized IEEE 39-bus system further demonstrates its efficiency in power networks grappling with significant renewables penetration. In summary, this integrated strategy presents a robust solution for modern power systems adapting to increasing renewable energy utilization.

## Introduction

### Background

Energy storage systems (ESSs) are becoming increasingly important as RESs become more prevalent in power systems. ESSs provide distinct benefits while also posing particular barriers in the field of energy storage$,$ engaging a critical role in spanning the gap between energy generation and demand while integrating renewable energy sources$,$ but necessitating careful technological advances in selection$,$ adaptability$,$ and incorporation into existing infrastructure. On the other hand$,$ it is essential to maintain equilibrium among power generation and demand in order to reinstate the power grid frequency to its desired orientation value. This task have been achieved by LFC^1^. In recent times$,$ the significance of LFC has grown substantially due to the expansion and complexity of interconnected power grids$,$ rising operational costs$,$ and the limitations of traditional units. These factors can potentially result in inadequate control performance. To address these challenges$,$ extensive research has been conducted considering LFC$,$ with a particular revolve around exploring the potential benefits of demand response. Numerous studies in the literature have aimed to investigate and comprehend how demand response^2^$,$ ESSs^3–5^$,$ RESs for example photovoltaic (PV) plants^6^$,$ flexible alternative current transmission system controllers^7^$,$ and wind turbine generators^8^.

### State-of-the-art literature review

The traditional approach to frequency control in power grids involves approximating the system as a linear model based on a specific operating condition without taking into account the dynamics of the generators. One commonly used method for frequency regulation is proportional-integral-derivative (PID) control$,$ which has been commonly applied in the ancient due to its merits such as affordability and simplicity. Nevertheless$,$ PID controllers are susceptible to vagaries in system constraints (e.g.$,$ system uncertainty and nonlinearities)^9^.However$,$ in recent years$,$ several control strategies$,$ such as fractional order controllers (FOCs) ^10,11^$,$ fuzzy logic control (FLC)^12^$,$ sliding mode control^13^$,$ artificial neural network^14^$,$ and model predictive control (MPC)^15^$,$ has been developed to address the frequency deviation problem.

In contrast, FOCs offer numerous advantages for power system stabilization$,$ providing a boarder extent of freedom and increased configuration flexibility. These characteristics make FOCs a perfect option for controlling power grids. Unlike traditional PID controllers$,$ FOCs require the fine-tuning of different types of poles$,$ like hyper-damped poles$,$ which expands the stable area and provides increased design flexibility for controllers. As a result$,$ FOCs reduce overshoot$,$ the settling time$,$ as well as undershoot values$,$ surpassing PID controllers and their variations. Furthermore$,$ wide-ranging researches have been conducted on the tender of FOCs in various studies$,$ with two common forms being firstly$,$ the title integral derivative (TID) form as well as the fractional order PID (FOPID)^4^. These FOCs have been thoroughly researched with the goal in order to boost the stability of frequency of today's electrical power grids^16–18^. While these controllers have demonstrated promising results in normal operating conditions.

In addition$,$ the fuzzy controller offers several key benefits$,$ including its straightforward implementation$,$ its ability to effectively respond to system variations$,$ as well as its ability to accommodate variations in the operational situations or system constraints through online updates of the controller constraints. These advantages stem from the controller's simplicity in execution and its high sensitivity to fluctuations in the power grid^19^. The initial application of FLC in addressing the LFC problem involved combining it with a PID controller$,$ as described in^20^. Furthermore$,$ a self-tuning fuzzy-PID controller has been introduced to improve the stability of the frequency of a two-area power grid^21^. Moreover$,$ the fuzzy-PID controller has been employed to stabilize the power structure’s frequency that incorporates multiple power sources^22^. According to the merits of FOCs and FLC$,$ the authors proposed the (fuzzy-PID) + (T ${I}^{\lambda }{D}^{\mu }$) controller to handle the LFC problematic in the analyzed two-area interconnected power grid. However$,$ the FLC utilizes various strictures such as inputs$,$ scaling factors$,$ membership functions$,$ and rule base. But$,$ there are no fixed rules to determine the values of these parameters. Typically$,$ trial-and-error methods have been employed to choice these values$,$ but they may not always yield optimal performance. To address this issue$,$ a meta-heuristic technique known as the Crayfish Optimization Algorithm (COA) has been used to select the optimal constraints of the proposed controller.

Lastly, multiple controllers in the LFC issue have been addressed through the application of optimization methods including a tracking methodology^23^ and aggregation strategies^24^ have been used to address. However, these prior techniques have certain downsides. To address the limitations of traditional optimization methods, which can get trapped in local minimums, require a high number of iterations, and are sensitive to initial conditions, researchers have focused their attention on meta-heuristic optimization methods, for example Coyote Optimization Algorithm^25^$,$ Dandelion Optimizer^26^$,$ Harris Hawks Algorithm^27^$,$ Archimedes Optimization Algorithm^28^$,$ Sine Cosine Algorithm^29^$,$ Ant Colony Optimization^30^$,$ Whale Optimization Algorithm^31^$,$ Grey Wolf Optimizer^32^$,$ Artificial Bee Colony Algorithm^33^$,$ Imperialist competitive Algorithm (ICA)^34^$,$ Quasi-oppositional Harmony Search^35,36^$,$ Quasi-opposition Pathfinder Algorithm^37^$,$ Chaotic Chimp Sine Cosine Optimization Algorithm^38^$,$ Quasi-oppositional Whale Optimization Algorithm^39^. These alternative approaches have gained attention as they offer a way to overcome these limitations and find optimal solutions more efficiently. By leveraging meta-heuristic optimization techniques, researchers can enhance the performance of LFC controllers and find more robust and effective parameter settings. Accordingly$,$ the authors propose appling the COA algorithm to determine the finest constraints of the considered controller. As, the considered COA method has been developed to address the limitations associated with traditional optimization techniques. The COA method offers advantages over conventional optimization algorithms by employing a gradient-free mechanism, which helps overcome the issue of getting stuck in local solutions. Moreover, the COA algorithm has the capability to find global solutions using a small number of search agents, further enhancing its superiority over other optimization methods^40^. In this work, it is the first time to apply the COA technique in handling the LFC issue.

From a different perspective, when there is a large impact as well as a high penetration of renewable energy, the frequency and tie line may be different for a long time, and oscillations may still occur. In this case, the LFC response may not be sufficient to absorb the frequency change due to the slowness and poor quality of the response. As a result, it is necessary to apply ESSs. However, different studies have applied different energy storage strategies with rapid rejoinder, like superconducting magnetic energy storage (SMES)$,$ fuel cell systems (FCSs)$,$ plug-in electric vehicles (PEVs)$,$ and redox flow batteries (RFBs)$,$ for addressing the LFC issue. The utilization of these devices has emerged as a new and intriguing research area^3,4,41–47^. Among these, RFB has shown its advantages over other electronic devices (such as SMES) due to its ability to operate at normal temperature, low loss and long service life. Consequently, RFBs is anticipated to be the most effective solution^48^. In a study described in ^49^$,$ the influence of RFBs and SMES units on enhancing the performance of a power grid was investigated. Simulation outcomes indicated that the incorporation of RFB units more effectively dampened dynamic responses in comparison to the SMES units. Additionally, the authors of demonstrated the superior performance of RFBs in combination with the Unified Power Flow Controller and Interline Power Flow Controller in Automatic Generation Control (AGC) on nonlinear power systems^43,50^. Furthermore, the stability is achieved in a nonlinear power system by incorporating integral control of the LFC model with RFBs storage and a Static Synchronous Series Compensator^51^.

### Research gap

Despite the pre-mentioned studies successfully achieved their objectives, these studies did not focus on the high incorporation of RESs in the presence of high load demand. For example, more than one previous study focused on conventional power plants and did not adequately consider the impact of RESs^7,22,41,42^. Nevertheless, the present study emphasizes high renewables penetration like wind and solar energy, which are commonly utilized in both areas of the power grid under examination. Additionally, numerous studies overlook the influence of diverse load perturbation shapes, which can be essential for evaluating the applied controller^25,26,28,30,31^. However, this study considered numerous load perturbation profiles like, step load disruptions (SLD)$,$ series SLD, and random load disruptions (RLD) which represent the forced outage of power plants or high change on the load demand. Moreover, most previous studies considered applying different types of ESSs without applying any controller to control their output^5,43^. However, this study consider the controlled ESSs to control the amount of the power which will injected to the overall system. The standard IEEE 39 buses test system is considered. Furthermore, the durability of the indicated controller is examined in this work by considering the challenge of the communication delay time (CDT). The purpose is to validate the effectiveness of the proposed controller in achieving stability and reliability in a practical power system setting. Furthermore, this paper’s main objective is to address the limitations uncovered in previously published investigations concerning the frequency stability problem. Therefore, Table 1 provides a clear overview of the variances between this study and other researches in the literature, emphasizing the unique contributions and approaches taken in this paper.

**Table 1:** Comparison of the present work's motivation to those of other published studies.

References	^5^	^7^	^16^	^22^	^49^	^51^	This work
Controller design	Combining TD-TI	Fuzzy fine-tuning approach	Cascaded TID	Fuzzy logic based self-tuning PID	PI	I	(fuzzy-PID) + (T ${{\text{I}}}^{\uplambda }{{\text{D}}}^{\upmu }$)
High penetration levels of RESs	√	×	×	×	×	×	√
Additional improvements incorporation	Considered PEVs	Considered series capacitors	Considered capacitor	not considered	Considered RFBs/ SMES	Considered RFBs/ SSSC	Considered CRFBs
Real-time validation considering various load patterns	×	×	×	×	×	×	IEEE-39 bus system/SLD
Real-time validation considering high RESs penetration	×	×	×	×	×	×	√
Real-time validation considering CDT	×	×	×	×	×	×	√

### Article motivations and contributions

The scope and contribution of this research can be summarized as follows:

- Proposing a concerted strategy based on enhanced LFC in coordination with ESSs (i.e., CRFBs) improves power grid stability during periods of disturbances in load and high RESs penetration. Where the proposed controller in LFC is (fuzzy-PID) + (T ${I}^{\lambda }{D}^{\mu }$) controller, whose superiority is confirmed by comparing its performance with other controllers like the (PID, FO-PID, PD-PI and FO-(PD-PI)).
- Considering the COA technique to select the optimal parameters of the considered controllers, according to its good optimization properties (i.e., balancing between exploration and exploitation) in order to enhance the performance of the analyzed power grid.
- The superiority of the proposed strategy is validated considering high RESs, load disturbances as well as CDT in considered hybrid power grid. Furthermore, the standard IEEE 39 buses is considered to confirm the superiority of the proposed strategy.

The remaining content of the manuscript is structured as follows: "Background" provides a detailed overview of the power grid under investigation, emphasizing its substantial penetration of renewable energy sources and the integration of controlled redox flow battery (CRFB) systems within the proposed strategy. "State-of-the-art literature review" describes the recommended approach, which involves the fuzzy-PID and (T ${I}^{\lambda }{D}^{\mu }$) controller, and formulates the problem under analysis. "Research gap" outlines the methodology employed for the crayfish optimization algorithm (COA) procedure. "Article motivations and contributions" presents the simulation results obtained from the aforementioned scenarios. Finally, in "Modeling of an investigated system", the study's conclusions and key findings are discussed and reviewed.

## Modeling of an investigated system

### The configuration of the considered system base

**Figure 1:**
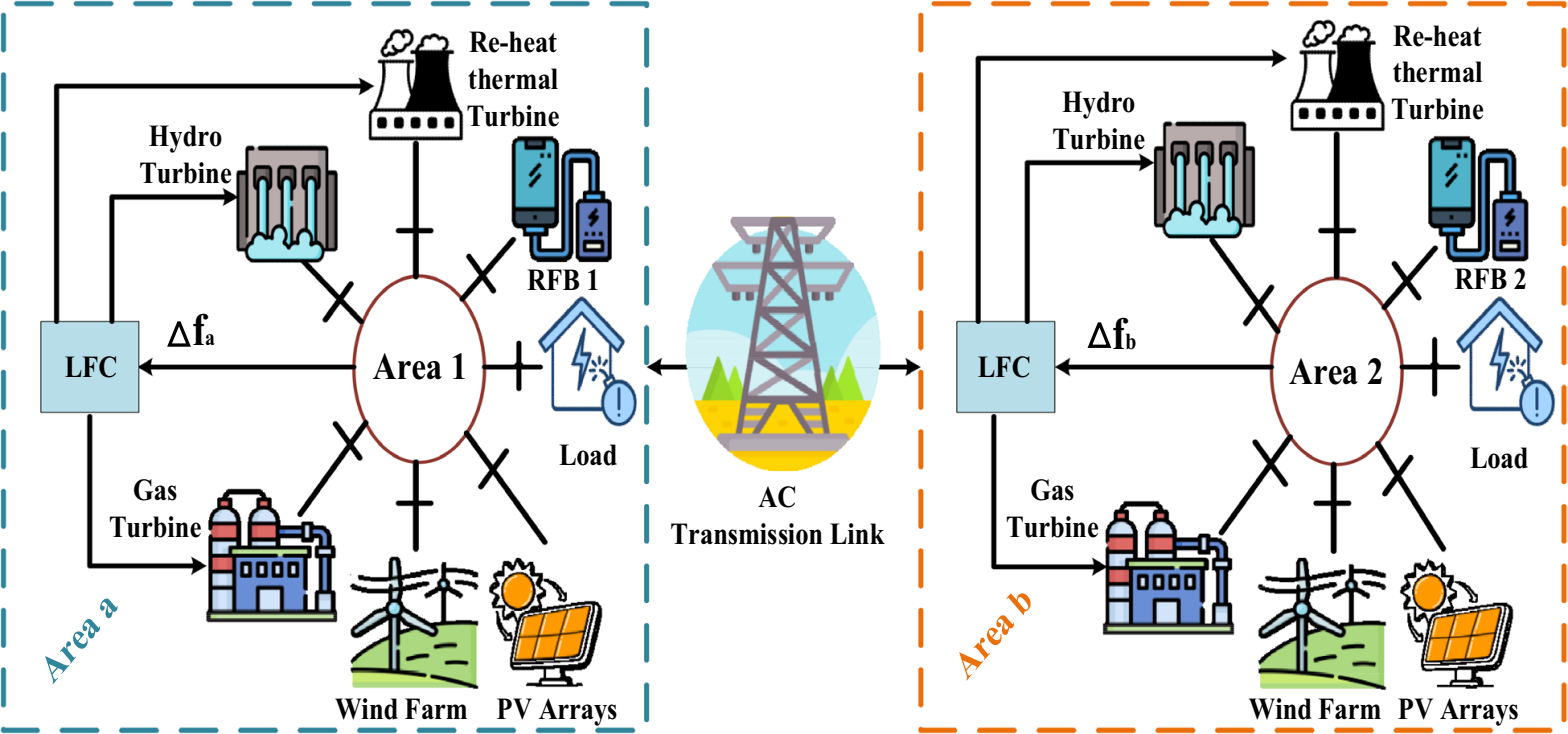
Schematic diagram of the considered grid.

**Figure 2:**
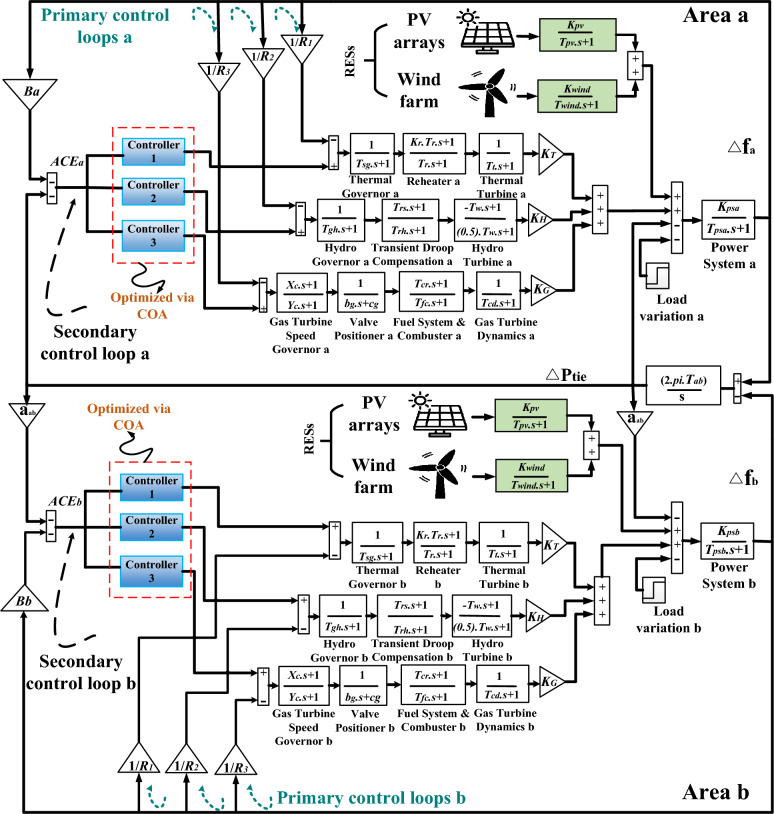
The linearized model of the Power grid under investigation.

The power grid that is mentioned in the literature is a two-identical area, with a three-traditional unit included within each area. A tie-line connecting the two areas is an AC tie-line. Thermal, hydro, and gas turbines are the three conventional units, which are dispersed throughout the two zones. In the present study, a 2000 $MW,$ total capacity electrical system is examined. In contrast, having a 1087 $MW,$ contribution, the thermal unit has the biggest capacity. The second-largest capacity, contributing 653 $MW,$ is possessed by the hydropower facility. And finally, 262 $MW,$ is contributed by the gas turbine capacity. Furthermore, Fig. 1 simplifies the diagram of the examined power grid, while the block diagram of the examined power grid is illustrated as Fig. 2. Next, each block's mathematical equation from Fig. 2 is presented in detail in Table 2. For the purpose of dealing with fluctuations in frequency in each area (e.g.$,$ area a -area b) and control the tie-line power flow between them, a (fuzzy-PID) + (T ${I}^{\lambda }{D}^{\mu }$) controller is proposed to be implemented for each power plant. The Area Control Error (*ACE)* serves as the input signal for the controller$,$ while the output signal represents the specific supplementary control action for each power plant. The aim of this procedure is to produce additional active power in order to improve the currently assessed grid performance. Appendix A provides comprehensive information about all the parameters related to the analyzed power grid, including their nominal values^52^. The following equations describe the formularies used to calculate the *ACEs* related to interconnected areas.

**Table 2:** The mathematical formulas regarding the considered power grid blocks.

Description of A block	Formulation of transfer functions
Power system a	$\frac{{K}_{psa}}{{T}_{psa}\cdot {\text{s}}+1}$
Power system b	$\frac{{K}_{psb}}{{T}_{psb}\cdot {\text{s}}+1}$
Hydro turbine	$\frac{-{T}_{w}\cdot {\text{s}}+1}{(0.5{).T}_{w}\cdot {\text{s}}+1}$
Transient droop compensation	$\frac{{T}_{rs}\cdot {\text{s}}+1}{{T}_{rh}\cdot {\text{s}}+1}$
Hydro governor	$\frac{1}{{T}_{gh}\cdot {\text{s}}+1}$
Reheater of thermal turbine	$\frac{{K}_{r}\cdot {T}_{r}\cdot {\text{s}}+1}{{T}_{r}\cdot {\text{s}}+1}$
Thermal governor	$\frac{1}{{T}_{sg}.{\text{s}}+1}$
Thermal turbine	$\frac{1}{{T}_{t}.{\text{s}}+1}$
Gas turbine's speed governor	$\frac{{x}_{c}\cdot {\text{s}}+1}{{Y}_{c}\cdot {\text{s}}+1}$
Gas turbine dynamics	$\frac{1}{{T}_{cd}\cdot {\text{s}}+1}$
Gas turbine's valve positioner	$\frac{1}{{b}_{g}\cdot {\text{s}}+{c}_{g}}$
Fuel system and combustor	$\frac{{T}_{cr}\cdot {\text{s}}+1}{{T}_{fc}\cdot {\text{s}}+1}$
Synchronizing coefficient	$\frac{{(2.pi.T}_{ab})}{{\text{s}}}$
Wind farm	$\frac{{K}_{Wind}}{{T}_{Wind}.{\text{s}}+1}$
PV Power plant	$\frac{{K}_{PV}}{{T}_{PV}.{\text{s}}+1}$

1 $${ACE}_{a}={\Delta {\text{P}}}_{\mathrm{tie a}-{\text{b}}}+{B}_{a}{\Delta {\text{f}}}_{{\text{a}}}$$

2 $${ACE}_{b}={\Delta {\text{P}}}_{\mathrm{tie b}-{\text{a}}}+{B}_{b}{\Delta {\text{f}}}_{{\text{b}}}$$

### Conceptualization of the wind farm

As highlighted in this analysis, the study's power grid is a hybrid system that contains a significant proportion of RESs, with a focus on wind generating facilities. The following formulae can be used to calculate wind output power^3,4^.

3 $${P}_{wt}= \frac{1}{2}\rho {A}_{T}{v}_{w}^{3}{C}_{p}\left(\lambda ,\beta \right)$$

The wind output power, denoted as ${P}_{wt}$ (in $MW$)$,$ is determined by various factors. These factors include the flounced sectioned area$, {A}_{T}$ (in ${m}^{2}$)$,$ the tip-speed ratio (TSR)$, \lambda ,$ the power coefficient$, {C}_{p},$ of the rotor blades, the air density, *ρ* (in $kg$/${m}^{3}$)$,$ the wind speed$, {V}_{W}$ (in $m$/$s$)$,$ and the blade pitch angle, *β*. The ${C}_{p}$ can be expressed as follow^53^:

4 $${C}_{p}\left({\lambda }_{i},\beta \right)=0.5\left({\lambda }_{i}-0.022{\beta }^{2}-5.6\right){\times e}^{-0.17{\lambda }_{i}}$$

5 $${\lambda }_{i}=\frac{3600\times R }{1609\times \lambda }$$

6 $$\lambda =\frac{{\omega }_{B}\times R}{{V}_{W}}$$

where ${\omega }_{B}$ specifies the angular velocity value of the blade (rad/sec).

**Figure 3:**
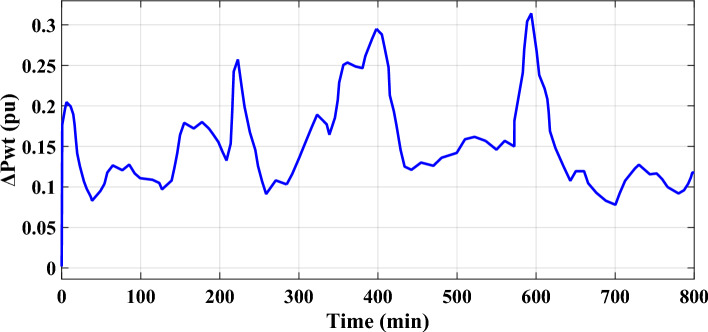
Energy generated by the Zafarana windmill station^53^.

The wind farm at Zafarana, Egypt, with a total installed capacity of 85 $MW,$ is included in this study. Additionally, Fig. 3 presents the real output power data for the Zafarana windmill. One may theoretically describe the characteristics of the transfer function, which depicts the behavior of the integrated induction generator in a wind power plant, as 0.3 s for the time constant (${T}_{wind}$) and a unity gain (${K}_{wind}$). Additionally, the overall capacity applied in this work is ten Zafarana wind farms, with a total capacity of 850 $MW$.

### Conceptualization of the photovoltaic (PV) power plant

This research also takes into account photovoltaic (PV) power plants, which generate electricity from solar energy besides utilized wind farms. Multiple variables, like the PV cell's surface area, the intensity of solar radiation, and the surrounding air temperature, affect a PV system's output power. For calculating the amount of energy generated by the module of PV in this current study, an equation is employed, taking into account these factors.

7 $$P={\eta }_{sc}{\tau }_{g}{\alpha }_{sc}RA [1-{\mu }_{sc}({T}_{sc}-{T}_{r})]$$

In the PV system, several parameters influence the output power calculation. These parameters include the cell reference efficiency denoted as ${\eta }_{sc},$ the entire solar cell's (SC) surface area denoted as $A$ (measured in ${m}^{2}$)$,$ the solar radiation denoted as $R$ (measured in W/${m}^{2}$)$,$ the transmissivity of the glass denoted as ${\tau }_{g},$ the absorptivity of the SC denoted as ${\alpha }_{sc},$ the PV cell efficiency thermal coefficient denoted as ${\mu }_{sc}$ (measured in $\frac{\%}{^\circ{\rm C} }$)$,$ the temperature of the SC denoted as ${T}_{sc}$ (measured in $^\circ{\rm C}$)$,$ then the reference temperature signified as ${T}_{r}$ (measured in $^\circ{\rm C}$). These parameters are used in a formula to determine the PV module's electrical power output.

**Figure 4:**
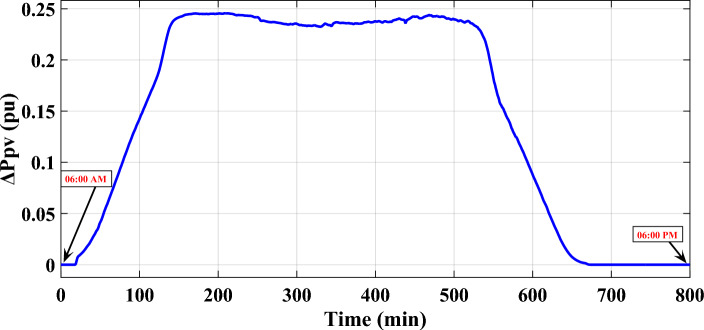
The exact PV power plant power generation^53^.

**Figure 5:**
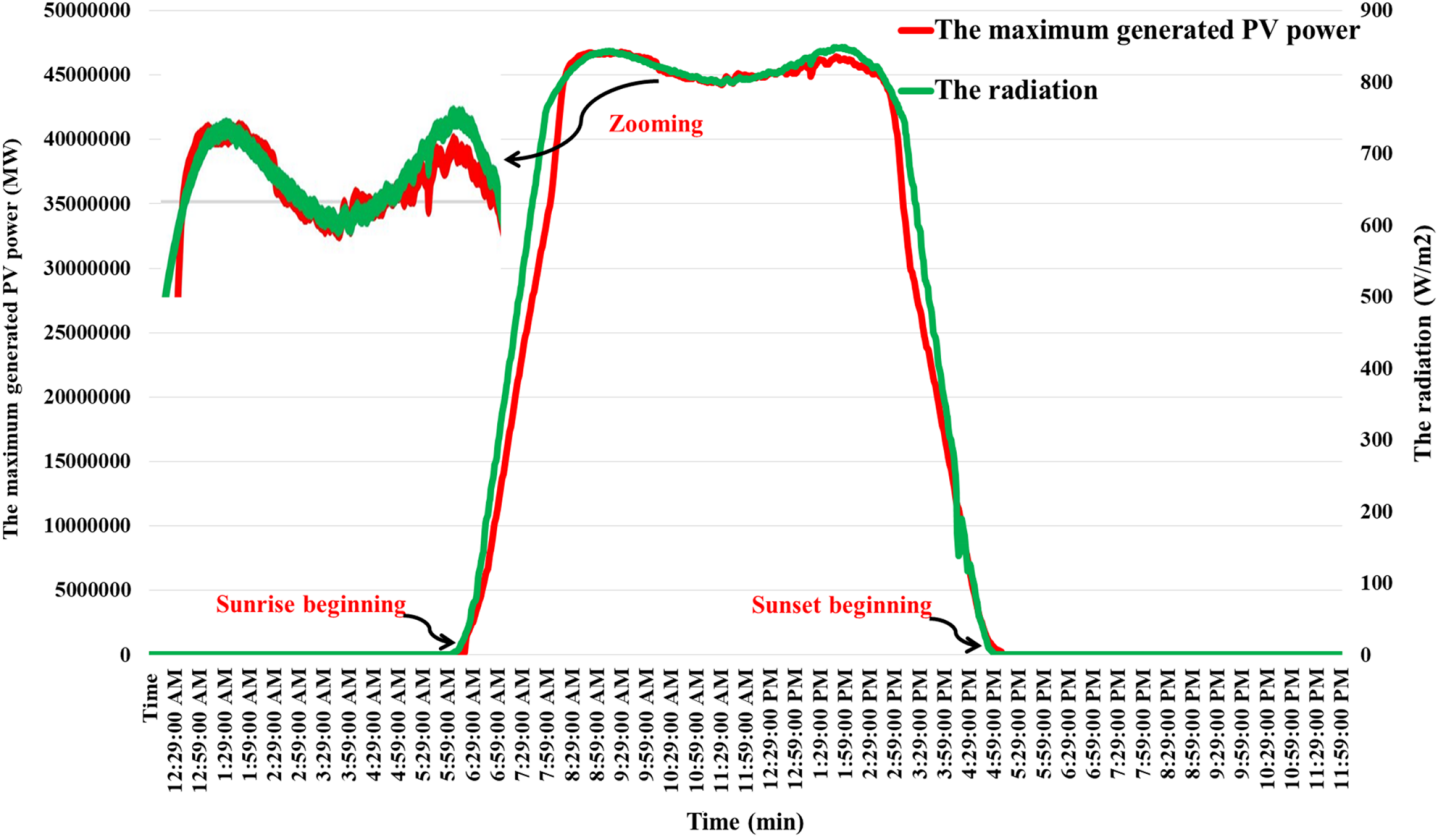
Radiation and power generation characteristics of the PV energy station.

**Figure 6:**
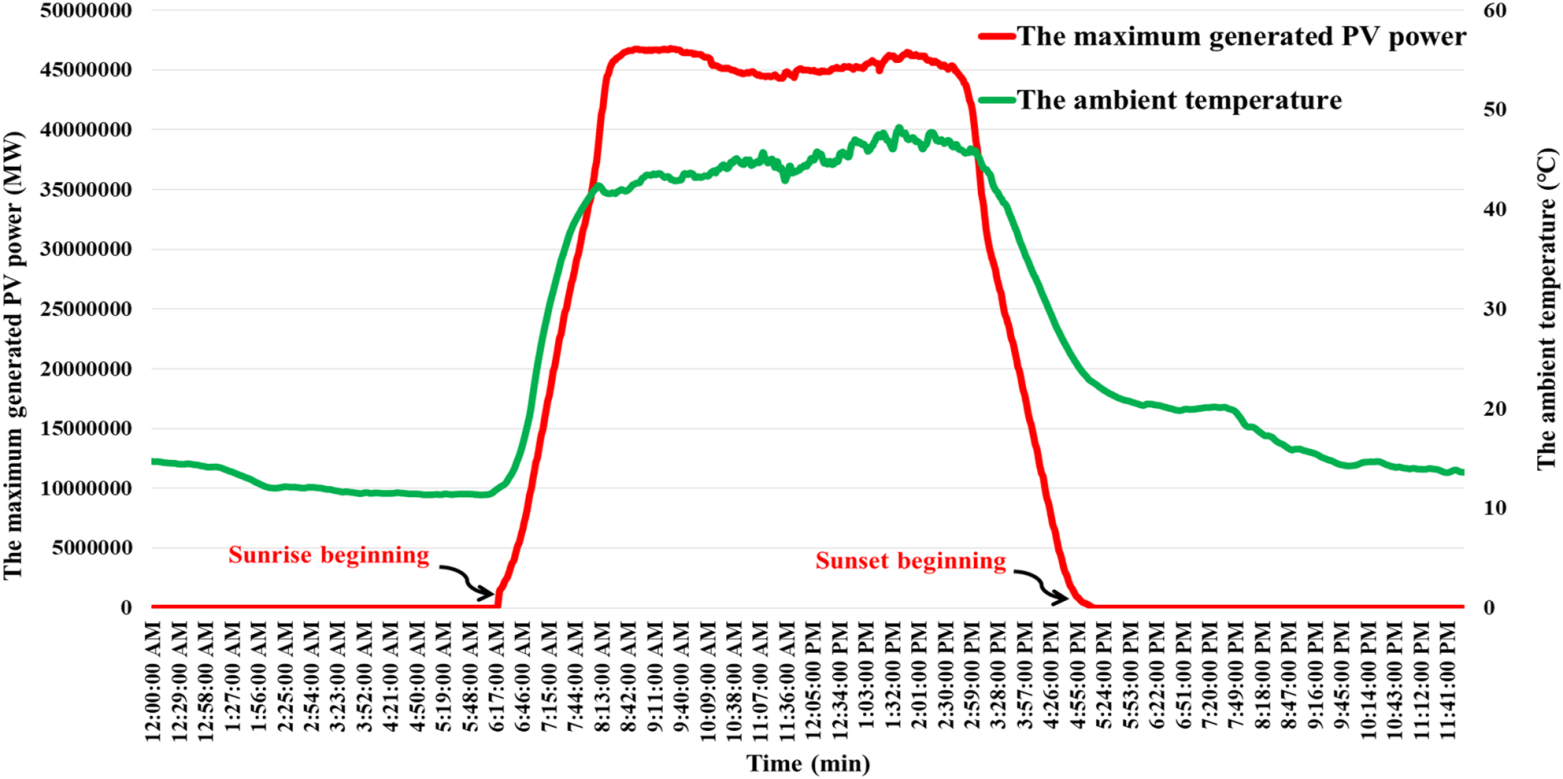
Power generation characteristics and ambient temperature of the PV energy station.

This study focuses on a specific power grid and examines a realistic 50 $MW$ PV system. The southern Egypt represents the location of this aforementioned realistic PV power plant. Whereas, the realistic PV power plant is modeled by a transfer function with 10 ms time constant (${T}_{PV}$) and a unity gain (${K}_{PV}$). The output power of this PV system is depicted in Fig. 4 illustrates the diagram of the output power produced by the PV system. Appendix B provides the results of the analysis of data gathered for identifying the correlation between brightness and PV temperature. Furthermore, the characteristics of the studied PV system are displayed in Figs. 5,6, which emphasize the associations between the produced active power, the values of brightness, the produced active power, and the temperature of the surroundings. Additionally, the overall capacity applied in this work is ten realistic PV power plants, with a total capacity of 500 $MW$.

### Controlled redox flow batteries (CRFBs) conceptualization

The CRFBs functions as an electrochemical power source that has the ability to be recharged. CRFBs can rapidly and efficiently save energy for the power network; additionally it can also employ the generator’s kinetic energy when requested^50^. It functions as a rapid energy compensating equipment for high load electrical consumption and additionally aids in frequency fluctuation suppression. The CRFBs has a broad range of applications and offers excellent features such as improved power quality, load compensation, higher capacity compared to conventional batteries, quick response time, and independence from self-discharge issues. During low load demand periods, the CRFBs stores and saves the energy that is equivalent to the demand variations. This stored energy can then be unrestricted back into the grid during load fluctuations, aiding in the elimination of power grid oscillations.

**Figure 7:**
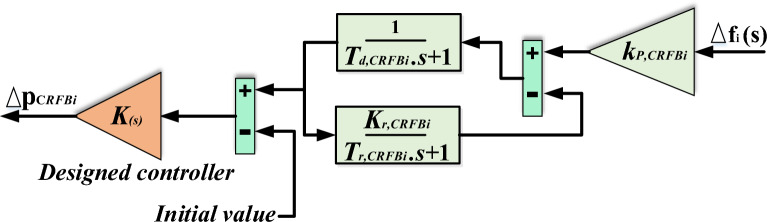
**C**RFBs unit’s transfer function model^45^.

In this study, a significant aspect of CRFBs modeling is the utilization of a comprehensive and realistic dynamic model of redox flow batteries, rather than the simplistic first-order transfer function models commonly used in several recent papers. The model incorporates the $\mathrm{\Delta f}$ (change in frequency) as an input. This paper presents a controller design that incorporates a feedback path to generate control signals for CRFBs. This design enables the CRFBs to respond efficiently to disturbances, resulting in improved performance. The designed controller, denoted as $K(s),$ is considered as a PID controller in this work. The relevant parameters of the CRFBs are provided in the Appendix B. Figure 7 illustrates the block diagram of CRFBs. Equation (8) indicates how the CRFBs unit is woven into the studied power grid^45^.

8 $$\Delta P_{CRFBi} = \left[ {k_{p,CRFBi} \Delta {\text{f}}_{i} \times \left( {\frac{{1 + {\text{s}}}}{{1 + k_{r,CRFBi} + {\text{s}}\left( {{\text{T}}_{r,CRFBi} + T_{d,CRFBi} } \right) + s^{2} T_{r,CRFBi} T_{d,CRFBi} }}} \right) - initial\,value} \right] \times K\left( {\text{s}} \right)$$

### Proposed control mechanism and problem description

In order to address the difficulties caused by the high renewables penetration, several load profile themes, and communication delays, it is crucial to design a reliable controller that can boost system performance under irregular situations. This paper recommends the utilizing of a (fuzzy-PID) + (T ${I}^{\lambda }{D}^{\mu }$) controller to alleviate the deviations resulting from the aforementioned factors. Structure-wise, both the TID and PID controllers are exactly the same, with one significant difference. Instead of the proportional component, the TID controller incorporates a tilted component with a transfer function of ${s}^{-(1/n)},$ where $n$ represents the FOCs. The TID controller has numerous benefits over a typical PID controller. It exhibits better disturbance rejection, simplified tuning process, and reduced sensitivity to variations in system parameters, resulting in improved dynamic response. Additionally, another type of FOCs called the fractional-order-integral-derivative (FO-ID) controller is available, which offers enhanced system performance and flexibility. Thus, this study replaced the second and third gains in TID controller by the FO-I and FO-D terms to attain the T ${I}^{\lambda }{D}^{\mu }$ controller. Where the frequency fluctuations of the area (${\Delta {\text{f}}}_{{\text{i}}}$) attends as the T ${I}^{\lambda }{D}^{\mu }$ controller’s input signal. Furthermore, the mathematical expression of T ${I}^{\lambda }{D}^{\mu }$ controller is expressed according to the next equation:

9 $$T{I}^{\lambda }{D}^{\mu }\left(s\right)= \frac{{K}_{ti,i}}{{S}^\frac{1}{n}}+\frac{{K}_{i,i}}{{S}^{\lambda }}+{K}_{d,i}{S}^{\mu }$$

The constraints of gain values$, {k}_{ti,i(T{I}^{\lambda }{D}^{\mu })},{k}_{i,i(T{I}^{\lambda }{D}^{\mu })},{k}_{d,i(T{I}^{\lambda }{D}^{\mu })},{n}_{(T{I}^{\lambda }{D}^{\mu })},{\lambda }_{(T{I}^{\lambda }{D}^{\mu })},$ and ${\mu }_{(T{I}^{\lambda }{D}^{\mu })}$ utilized in this work in both areas of the considered power grid are expressed as follows:

10 $${k}_{ti,{i(T{I}^{\lambda }{D}^{\upmu })}^{max}} \ge {k}_{ti,i\left(T{I}^{\lambda }{D}^{\upmu }\right)} \ge {k}_{ti,{i(T{I}^{\lambda }{D}^{\upmu })}^{min}}$$

11 $${k}_{i,{i(T{I}^{\lambda }{D}^{\upmu })}^{max}} \ge {k}_{i,i\left(T{I}^{\lambda }{D}^{\upmu }\right)} \ge {k}_{i,{i(T{I}^{\lambda }{D}^{\upmu })}^{min}}$$

12 $${k}_{d,{i(T{I}^{\lambda }{D}^{\upmu })}^{max }} \ge {k}_{d,i\left(T{I}^{\lambda }{D}^{\upmu }\right)} \ge {k}_{d,{i(T{I}^{\lambda }{D}^{\upmu })}^{min}}$$

13 $${n}_{{(T{I}^{\lambda }{D}^{\upmu })}^{max}} \ge {n}_{\left(T{I}^{\lambda }{D}^{\upmu }\right)} \ge {n}_{{(T{I}^{\lambda }{D}^{\upmu })}^{min}}$$

14 $${\lambda }_{{(T{I}^{\lambda }{D}^{\upmu })}^{max}} \ge {\lambda }_{\left(T{I}^{\lambda }{D}^{\upmu }\right)} \ge {\lambda }_{{(T{I}^{\lambda }{D}^{\upmu })}^{min}}$$

15 $${\upmu }_{{(T{I}^{\lambda }{D}^{\upmu })}^{max}} \ge {\upmu }_{\left(T{I}^{\lambda }{D}^{\upmu }\right)} \ge {\upmu }_{{(T{I}^{\lambda }{D}^{\upmu })}^{min}}$$

where $i$ refers scheme of the suggested controller for each of the three mentioned traditional power plants; thus, ($i$=1, 2, 3). The gain values (${k}_{ti,i(T{I}^{\lambda }{D}^{\mu })},{k}_{i,i(T{I}^{\lambda }{D}^{\mu })},{k}_{d,i(T{I}^{\lambda }{D}^{\mu })},{n}_{(T{I}^{\lambda }{D}^{\mu })},{\lambda }_{(T{I}^{\lambda }{D}^{\mu })},$ and ${\mu }_{(T{I}^{\lambda }{D}^{\mu })}$) are chosen between [0, 10]$,$ ${n}_{(T{I}^{\lambda }{D}^{\mu })}$ is set among the range [1, 10]$,$ the fractional derivative (${\mu }_{(T{I}^{\lambda }{D}^{\mu })}$) and fractional integrator (${\lambda }_{(T{I}^{\lambda }{D}^{\mu })}$) orders are chosen among the range [0, 1].

**Figure 8:**
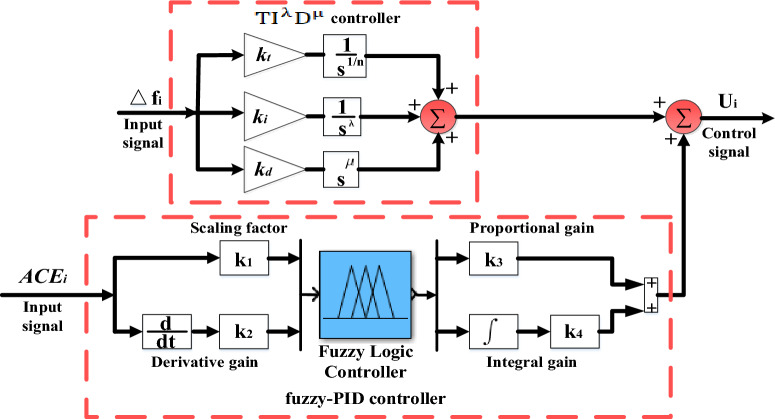
Considered (fuzzy-PID) + (T ${I}^{\lambda }{D}^{\mu }$) controller arrangement.

Additionally, Fig. 8 illustrates the proposed controller structure for the three considered generating units. Where, the considered controller has dual inputs: the *ACE* value and the derivative value of *ACE.* The scalability variables for the inputs are symbolized as K_1_ and K_2_, while the scalability variables for the output are symbolized as K_3_ and K_4_. Traditionally, determining these scaling factors has been a challenge, often relying on trial-and-error methods. This can make obtaining optimal parameter values that improve system performance difficult. However, this study overcomes this limitation by designing the considered (fuzzy-PID) + (T ${I}^{\lambda }{D}^{\mu }$) controller. Furthermore, the COA algorithm is used to determine these optimized values, where, the fuzzy membership function can be either triangular or Gaussian shape. In practical applications of electrical systems, triangular membership is often preferred due to its computational efficiency and simplicity. It is a first-order function of mathematics that decreases computational load. Triangular membership functions are typically used with PID controllers and exhibit abundantly symmetric characteristics in both output as well as input. Furthermore, the mathematical expression of (fuzzy-PID) controller is expressed according to the following equation:

16 $$fuzzy-PID\left(s\right)={K}_{1,i}+{K}_{3,i}+\frac{{K}_{4,i}}{s}+{K}_{2,i}s$$

The constraints of gain values$, {K}_{1,i}, {K}_{2,i},{K}_{3,i},$ and ${K}_{4,i}$ utilized in this work in both areas of the analyzed power grid are expressed as follows:

17 $${k}_{1,{i(fuzzy-PID)}^{max}} \ge {k}_{1,i\left(fuzzy-PID\right)} \ge {k}_{1,{i(fuzzy-PID)}^{min}}$$

18 $${k}_{3,{i(fuzzy-PID)}^{max}} \ge {k}_{3,i\left(fuzzy-PID\right)} \ge {k}_{3,{i(fuzzy-PID)}^{min}}$$

19 $${k}_{4,{i(fuzzy-PID)}^{max}} \ge {k}_{4,i\left(fuzzy-PID\right)} \ge {k}_{4,{i(fuzzy-PID)}^{min}}$$

20 $${k}_{2,{i(fuzzy-PID)}^{max}} \ge {k}_{2,i\left(fuzzy-PID\right)} \ge {k}_{2,{i(fuzzy-PID)}^{min}}$$

The gain values (${k}_{1,i(fuzzy-PID)},{k}_{2,i(fuzzy-PID)},{k}_{3,i(fuzzy-PID)},$ and ${k}_{4,i(fuzzy-PID)}$) are chosen between [0, 10].

**Figure 9:**
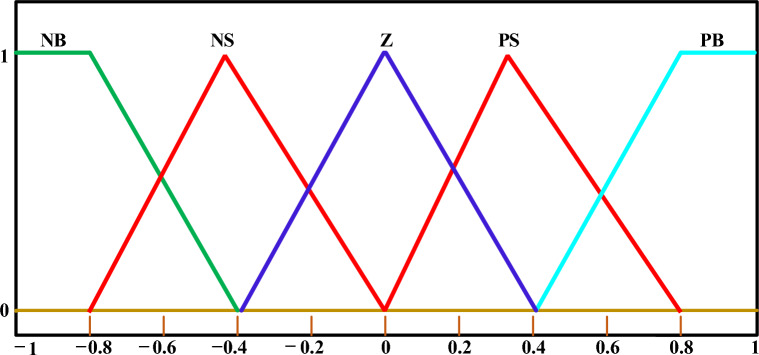
The triangular membership functions of the FLC's input and output.

The decision about the choice of fuzzy control settings is influenced by the specific characteristics of the system under consideration as well as the designer's expertise. The range of fuzzy function memberships is determined by predicting the system's input and output discourse universes. Typically, a decision-maker defines the acceptable risk level for the output and input variables. In this study, the range intervals are selected between -1 and 1, as deviations beyond this range are not necessary to achieve system stability. Symmetric triangular membership functions with 50% overlap are recommended, and a tuning procedure can be applied to adjust the spread and overlap as needed to achieve satisfactory results. In this study, the fuzzy-PID controller in this work utilizes five triangular membership functions: negative big (NB)$,$ negative small (NS)$,$ zero (Z)$,$ positive small (PS)$,$ and positive big (PB). These membership functions, depicted in Fig. 9, are utilized for outputs as well as inputs. Consequently, the fuzzy-PID controller requires 25 rules to generate fuzzy outputs, which play a crucial role in its performance. These rules are presented in Table 3. Also, the arrangement of the considered combination of (fuzzy-PID) and (T ${I}^{\lambda }{D}^{\mu }$) controller is depicted in Fig. 8. According to various published studies, the load frequency controller's input signal is usually *ACE*. In this study, the authors proposed (fuzzy-PID) + (T ${I}^{\lambda }{D}^{\mu }$) controller, which combines two concepts. The first (fuzzy-PID) controller term receives the *ACE* signal as an input. The extra (T ${I}^{\lambda }{D}^{\mu }$) controller term employs the $\Delta {\text{f}}$ signal to improve system performance. It is obviously true that the total arrangement of the proposed controller demonstrates superior rejection of existing disturbances by mitigating all low- and high-frequency disturbances^4,6^.

**Table 3:** Rule base of the FLC.

$ACE$	$dACE/dt$				
	NB	NS	Z	PS	PB
NB	NB	NB	NB	NS	Z
NS	NB	NB	NS	Z	PS
Z	NB	NS	Z	PS	PB
PS	NS	Z	PS	PB	PB
PB	Z	PS	PB	PB	PB

In this study, the authors propose the utilization of the integral time absolute error (*ITAE*) criteria as a means for minimizing the value of the cost function. Optimizing development is improved by adding the element of a time term to the *ITAE* criteria and the system constancy will be more enhanced compared to using the integral of squared error (*ISE*) criteria. Increased efficacy in error reduction is achieved by multiplying the factor of time by the integral of the absolute error. The *ITAE* principles can be expressed as follows:

21 $$J\hspace{0.17em}=\hspace{0.17em}ITAE\hspace{0.17em}=\hspace{0.17em}\underset{0}{\overset{Tsim}{\int }}t. \left[\left|{\Delta {\text{f}}}_{{\text{a}}}\right|+\left|{\Delta {\text{f}}}_{{\text{b}}}\right|+\left|{\Delta {\text{p}}}_{{\text{tie}}}\right|\right]. dt$$

Where *J* denotes the value of proposed cost function which seeks to be the lowest possible value in this work$, \left|{\Delta {\text{f}}}_{\mathrm{a }}\right|$ represents the area-a frequency waveform's absolute error$, \left|{\Delta {\text{f}}}_{{\text{b}}}\right|$ represents the area-b frequency waveform's absolute error$, \left|{\Delta {\text{p}}}_{{\text{tie}}}\right|$ represents the tie-line’s power flow change absolute error$, Tsim$ denotes the overall simulation runtime, and the variable $dt$ symbolizes the error signal sampling period throughout the simulation procedure.

### The crayfish optimization algorithm (COA)

The Crayfish Optimization Algorithm (COA) principles which is proposed by Heming et.al is discussed in detail in this section^40^. Foraging, competition, and summer resort behaviors are the three crayfish behaviors that the COA simulates. To equilibrium the algorithm's exploration and exploitation features, these behaviors are classified into three stages of actions (i.e.$,$ summer resort, competition, and foraging). The summer resort stage symbolizes the COA's exploring phase, while the competition and foraging stages indicate the exploitation phase. The temperature has an effect on the algorithm's exploration and exploitation. The initial stage of the algorithm defines a crawfish colony, denoted as $X,$ where ${X}_{i}$ indicates a solution and describes the position of the $ith$ crayfish. (${X}_{i}$=$\left\{{X}_{i,1},{X}_{i,2},{X}_{i,3}\dots {X}_{i,dim}\right\},$ dim is the dimension which denotes the optimization problem’s characteristic quantity. Random constants that represent the ambient temperature have an influence on individual’s behavior. When the temperatures increase, the COA moves into the stage of summer resort or the competition stage. New solutions are updated at the summer resort stage depending on the position of an individual $,{ X}_{i}$ and the position of cave $,{ X}_{shade}$. When the ambient temperature is appropriate and warm enough, the COA begins the foraging stage. During this stage, the best location, likewise regarded as an optimal solution, reveals the location of food. The ${fitness}_{i}$ value of the current solution (gained through ${X}_{i}$) and the optimal solution ${fitness}_{food}$ value (gained as a result of the optimal solution) define the size of the food. During foraging, crayfish obtain new solutions by considering their position$, {X}_{i}$ a food intake constant $p,$ and the update of the food position X _*food*_. If crayfish want to eat food that is excessively large for them, they will first break it down with their claw foot before alternating between their second and third walking feet. Overall, the COA simulates crayfish behavior by incorporating exploration and exploitation through temperature regulation, updating solutions based on individual and optimal positions, and considering the size and consumption of food.

This subsection summarizes the methodology of the COA algorithm:

#### Initialization process of COA

This study proposes a controller with both lower and upper limits for parameters. To accomplish the global goal, the population of the COA method is formed between these boundaries using main mathematical procedures. The population of the COA method can be expressed using Eq. (22).

22 $$X_{i,j} = rand \times (UB_{j} - LB_{j} ) + LB_{j}$$

Where ${X}_{i,j}$ represents where the individual $i$ is located in the $j$ dimension$, {LB}_{j}$ represents the $jth$ dimension's lower bound$, {UB}_{j}$ represents the $jth$ dimension's upper bound, and $rand$ is a random number.

The COA method starts by generating candidate solutions ($X$) randomly. In each iteration, the obtained solution that is closest to the global goal (target) represents the optimal solution. The obtained solutions’ positions are indicated in the next matrix.

23 $$X=\left[\begin{array}{cc}\begin{array}{cc}{X}_{\mathrm{1,1}}& {X}_{1,j}\\ {X}_{i,1}& {X}_{i,j}\end{array}& \begin{array}{cc}\cdots & {X}_{1,dim}\\ \cdots & {X}_{i,dim}\end{array}\\ \begin{array}{cc}\vdots & \vdots \\ {X}_{N,1}& {X}_{N,j}\end{array}& \begin{array}{cc}\ddots & \vdots \\ \dots & {X}_{N,dim}\end{array}\end{array}\right]$$

where $X$ refers the initial population position$, N$ refers the population number$, dim$ refers the population dimension.

The fitness solution, which represents the solution with the best fitness value among the obtained solutions, is calculated as following:

24 $${f}_{fitness}= {[f1 f2 f3\dots \dots \dots \dots {f}_{N}]}^{T}$$

#### Definition of the crayfish temperature and its intake

The behavior of crayfish is influenced by changes in temperature, which determine the stage they enter. Temperature is determined by Eq. (25). When the temperature exceeds 30 °C, crayfish will seek out cooler areas for taking a summer vacation. Crayfish will begin to forage when the temperature is appropriate. Temperature influences the amount of food consumed by crayfish. Crayfish prefer temperatures between 15 °C and 30 °C for feeding, with 25 °C being the most favorable. Equation (26) provides the mathematical representation of the crayfish intake.

25 $$Temp=rand \times 15+ 20$$

In this equation, "$Temp$ " denotes the temperature of the crayfish's environment.

26 $$p= {C}_{1} \times \left(\frac{1}{\sqrt{2\times \pi }\times \sigma }\times {\text{exp}}(\frac{{-\left(Temp-\mu \right)}^{2}}{2{\sigma }^{2}})\right)$$

Among them, $\sigma$ and ${C}_{1}$ is used to regulate crayfish intake at various temperatures, while $\mu$ refers to the optimal crayfish temperature.

#### The exploration stage (summer resort stage)

When the temperature exceeds 30 °C, it is considered too high, and crayfish will choose to seek refuge in caves to take the period of summer vacation. The cave ${X}_{shade}$ is identified as listed below:

27 $$\,X_{shade} = \,(X_{G} + X_{L} )/{2}$$

In this scenario$, {X}_{G}$ demonstrates the optimal position attained so far throughout iterations, while ${X}_{L}$ represents the current population’s optimal position.

The competition among crayfish for caves is a random occurrence. When a randomly generated number $rand$<0.5, it means that there aren't any other crayfish fighting for caves. In such instances, the crayfish are going to move directly to the cave for their break during the summer vacation. This behavior is governed by Eq. (28).

28 $${X}_{i,j}^{t+1}= {X}_{i,j}^{t}+ {C}_{2} \times rand\times \left({X}_{shade}- {X}_{i,j}^{t}\right)$$

Where $t$ refers the current iteration number, and t+1 refers the number of the next generation iteration,C_2_ refers the decreasing curve, as proved by Eq. (29).

29 $$C_{2} = 2 - (t/T)$$

where $t$ indicates the maximum iterations' number.

Crayfish's goal at the summer resort stage is to go close to the cave, which represents the optimal solution. Consequently, crayfish will move towards the cave in an effort to get closer to the optimal solution. Individuals become closer to the best solution through this process, which also improves the COA algorithm's exploitation capability, thus allowing for faster convergence.

#### The exploitation stage (competition stage)

When the temperature exceeds 30 °C and $rand$≥0.5, it indicates that other crayfish are also captivated by the cave. In this situation, they will engage in a competitive fight to gain access to the cave. As per Eq. (30), the crayfish competes over the cave:

30 $${X}_{i,j}^{t+1}= {X}_{i,j}^{t} - {X}_{z,j}^{t} + {X}_{shade}$$

Where $z$ sums up the crayfish's arbitrarily individual, as demonstrated in Eq. (31):

31 $$z= round(rand \times \left(N-1\right)+ 1$$

Crayfish compete over the cave during the competition stage. Each crayfish, denoted as ${X}_{i},$ adjusts its position depending on where another crayfish is located$, {X}_{z}$ as described in Eq. (30). This adjustment of positions expands the search range of the COA algorithm, enhancing its exploration ability.

#### The exploitation stage (foraging stage)

When the $Temp$ ≤ 30 °C, it seems to be appropriate for feeding crayfish. In this case, the crayfish will keep moving in the direction of the food supply. Once they find the food, crayfish will assess its size. Crayfish will use their claws to rip apart food if it is deemed to be excessively large. Then, they will alternately use their two remaining walking feet to consume their food. Food location, denoted as ${X}_{food},$ is defined as follows:

32 $${X}_{food}= {X}_{G}$$

The food size *Q *is defined as:

33 $$Q= {C}_{3} \times rand \times ({fitness}_{i}/{fitness}_{food})$$

Here$, {C}_{3}$ refers the factor of food, which represents the largest food size and has a constant value of 3. "${fitness}_{i}$" refers the $ith$ crayfish’s fitness value, while “*fitness*_*food*_” refers the food location’s fitness value. The crayfish uses the largest food's quantity as a benchmark for determining the size of food. When $Q$ surpasses (${C}_{3}$+1)/2, it implies a large amount of the food. Here, the crayfish is going to utilize their first claw foot for ripping the food. The mathematical equation representing this action is provided as follows:

34 $$X_{food} = {\text{exp}}\left( {\tfrac{ - 1}{Q}} \right) \times X_{food}$$

This mechanism enables crayfish to adapt their feeding behavior depending on the size of the food available, ensuring efficient consumption and utilization of resources.

When the food becomes crumbly and smaller, the crayfish will pick it up with their second and third paws alternatively and put it in their mouth. A sine and cosine function combination is utilized to describe this alternating process. Furthermore, the crayfish's food intake is related to the food they get. Foraging is calculated as follows:

35 $${X}_{i,j}^{t+1}= {X}_{i,j}^{t}+ { X}_{food } \times p \times ({\text{cos}}\left(2\times \pi \times rand\right)-{\text{sin}}(2\times\uppi \times rand)$$

When $Q$ ≤ (${C}_{3}$+1)/2, the crayfish only needs to proceed forward to the food and devour it immediately. The equation for this behavior is provided as follows:

36 $${X}_{i,j}^{t+1}=\left({X}_{i,j}^{t} - {X}_{food}\right)\times p+ p \times rand \times {X}_{i,j}^{t}$$

**Figure 10:**
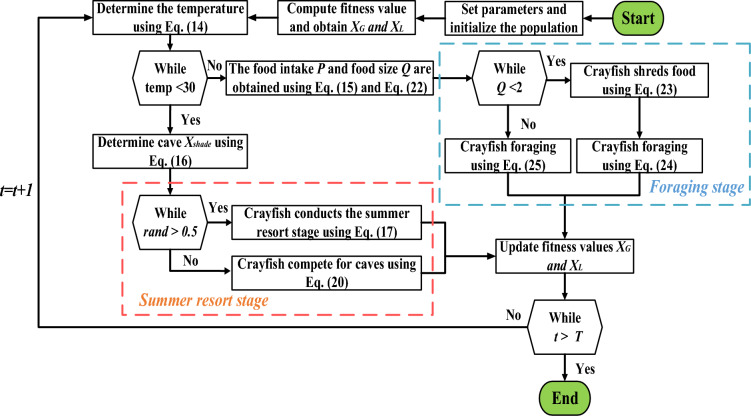
The flow chart of the COA algorithm^40^.

Crayfish use a variety of feeding strategies during the foraging stage, which are determined by their food size, denoted as $Q$. The food location ${X}_{food}$ represents the optimal solution in this context. If the food $Q$'s size is appropriate for crayfish feeding, they will approach the food and consume it. However, if $Q$ is too large, it shows that there is a significant difference between the optimal solution and the crayfish. In such cases$, {X}_{food}$ needs to be adjusted to reduce the difference as well as moving it up close to the food. Furthermore, the randomness of the crayfish food intake enhancement algorithm is under executive control. This ensures that the crayfish can approach the optimal solution more effectively during the foraging stage. Through this process, the COA algorithm gradually converges towards the optimal solution, enhancing its exploitation ability and achieving good convergence performance. Here, Fig. 10 depicts the COA method’s flowchart.

### Ethical approval

This article does not contain any studies with human participants or animals performed by any of the authors.

## Execution and results discussion

In this work, a supplementary control scheme called (fuzzy-PID) + (T ${I}^{\lambda }{D}^{\mu }$) is proposed as well as implemented for the analyzed power grid. According to the merits of fuzzy logic and fractional order schemes, the proposed controller outperforms superior rejection of existing disturbances by dampening all low- and high-frequency disruptions. This present study addresses the presence of high renewables penetration in the power grid. Multiple scenarios are scrutinized to evaluate how well the suggested control approach coordinates several different controllers. The proposed algorithm's performance is additionally assessed in comparison to different techniques. To optimize the suggested controller, the COA technique is employed to alleviate system frequency excursions. The simulations are conducted using MATLAB/SIMULINK® program (R2016a) on a computer with specific hardware specifications. For optimizing the proposed control structure, an m-file containing the original COA algorithm code and paired with the stipulated power grid model is employed. The several simulation scenarios are executed on a PC with the following specifications: 8.00 GB of RAM with a 2.60 GHz Intel Core i5 processor. Furthermore, the frequency stability assessing takes into consideration varied operational conditions in the scenarios listed below:

- *Scenario A* Evaluating the analyzed power grid’s performance with different varied load pattern profiles.
- *Scenario B* Evaluating the examined power grid’s performance involving high renewables penetration, aiming to examine the grid's capability to handle this issue.
- *Scenario C* Evaluating the suggested controller's robustness in real-time for stability and reliability using the  IEEE-39 bus system. The validation includes considering the impact of SLD.
- *Scenario D* Evaluating the efficacy of the recommended controller utilizing a real-time IEEE-39 bus system, considering high renewables penetration within the system, to assess its effectiveness with the goal to attain dependability and stability.
- *Scenario E* Evaluating the performance of the proposed controller under varying CDT conditions before and after the LFC in the IEEE-39 bus system. This evaluation also considers high renewable energy penetrations.
- *Scenario F* Evaluating the proposed strategy’s effectiveness by comparing the effectiveness of the IEEE 39 buses system with and without the proposed strategy, considering high renewables penetration.
- *Scenario G* Stability analysis of the proposed (fuzzy-PID) + (T ${I}^{\lambda }{D}^{\mu }$) controller.

**Figure 11:**
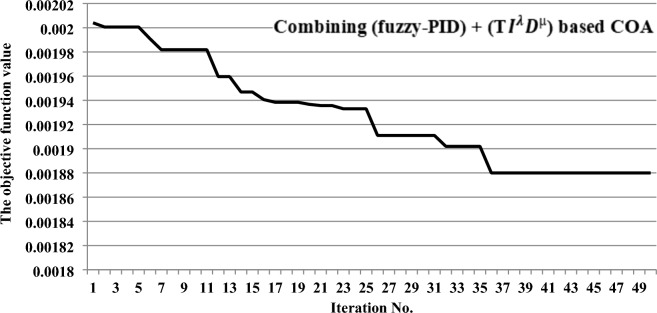
Obtained convergence curve of the suggested controller tuned by the COA method.

When optimizing the proposed (fuzzy-PID) + (T ${I}^{\lambda }{D}^{\mu }$) controller using the COA algorithm, certain arrangements should be considered. These include employing 30 search agents and performing 50 iterations. Figure 11 shows the obtained convergence curve, which provides insights into the investigated power grid’s performance with the recommended COA-based controller. It should be noted that the presented convergence curve in the figure is obtained assuming a 1% SLD occurring to area-a at t = 10 s within the examined power grid, without considering a high renewables penetration. Whereas, the behavior of proposed controller based on COA is summarized as beginning with a value of the objective function near 0.00201 and dropping with each attempt until it achieves a value near 0.00188.

*Scenario A* Evaluating the analyzed power grid’s performance with different varied load pattern profiles.

The following scenario is intended to provide an initial comparison for accurately verifying the suggested controller’s efficiency and algorithm in achieving frequency stabilization of the analyzed power grid. To conduct a more detailed analysis, a comparison is made among various aforementioned published controllers.

**Figure 12:**
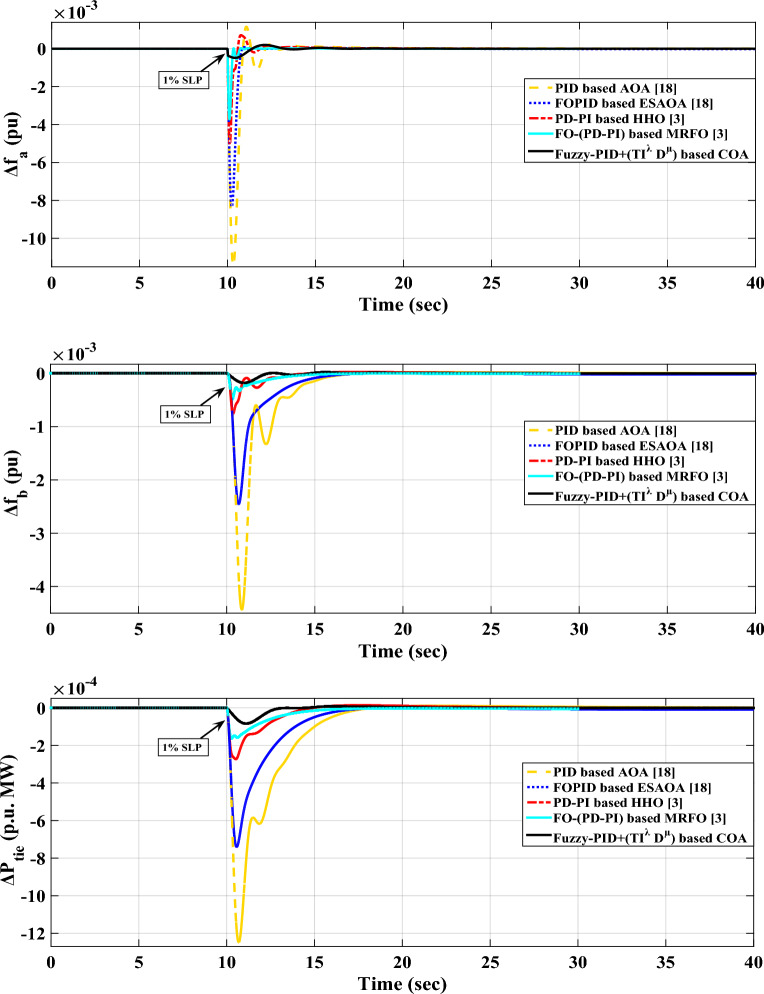
LFC controllers' dynamic responses to step load disruption for Case A.1.

*Case A.1* In this case, it is assumed that area-a of the assessed power grid includes a step load. The purpose is to assess the effectiveness of the COA algorithm in addressing the frequency stability issue through the use of the supplementary control loop. Assuming a step load of 1% magnitude at t = 10 s, representing either the disconnection of generation power plants or the disconnection of a variety of electrical loads. The obtained controller parameters for the proposed controller and the other mentioned controllers are presented in Table 4. Additionally, the system's performance is depicted in Fig. 12.

**Table 4:** Optimal values of various optimized controllers.

Controller structures	Thermal unit	Hydro unit	Gas unit	Objective function value (*ITAE*)
PID-optimized via AOA^18^	${k}_{p}$= 10$, {k}_{i}$= 1.5975$, {k}_{d}$= 2.7449	${k}_{p}$= 1.5975$, {k}_{i}$= 0.0837$, {k}_{d}$= 0.0875	${k}_{p}$= 10$, {k}_{i}$= 10$, {k}_{d}$= 1.2779	0.189
FOPID-optimized via ESAOA^18^	${k}_{p}$= 8.9645$, {k}_{i}$= 9.9979$, {k}_{d}$= 9.7397, λ = 0.7266, µ = 0.8519	${k}_{p}$= 6.6278$, {k}_{i}$= 9.1570$, {k}_{d}$= 4.631, λ = 0.587, µ = 0.0887	${k}_{p}$= 9.7594$, {k}_{i}$= 9.9997$, {k}_{d}$= 7.976, λ = 0.9159, µ = 0.3775	0.1233
PD-PI- optimized via HHO^3^	${k}_{p1}$= 5, ${k}_{d1}$ = 5, ${k}_{p2}$ = 5, ${k}_{i1}$ = 5	${k}_{p1}$= 5$, {k}_{d1}$ = 0.4447$, {k}_{p2}$ = 5, ${k}_{i1}$ = − 2.6257	${k}_{p1}$= 5$, {k}_{d1}$ = − 5$, {k}_{p2}$ = 5 $,$ ${k}_{i1}$ = 5	0.04312
FO-(PD-PI)- optimized via MRFO^3^	${k}_{p1}$= 8.4889$, {k}_{d1}$ = 4.7798, µ = 0.888$, {k}_{p2}$ = 3.3705$, {k}_{i1}$ = 9.1858, λ = 0.63405	${k}_{p1}$= 8.3087$, {k}_{d1}$ = 7.8085, µ = 0.4372$, {k}_{p2}$ = 7.6906$, {k}_{i1}$ = 9.3538, λ = 0.13835	${k}_{p1}$= 0.4128$, {k}_{d1}$ = 4.8394, µ = 0.6828$, {k}_{p2}$ = 1.8088$, {k}_{i1}$ = 0.95754, λ = 0.82713	0.03477
Fuzzy-PID + (T ${I}^{\lambda }{D}^{\mu }$)- optimized via COA	${k}_{1}$= 10$, {k}_{2}$ = 9.9229$, {k}_{3}$ = 6.923$, {k}_{4}$ = 10$, {k}_{t}$ = 10$, {k}_{i}$ = 9.9827$, {k}_{d}$ = 5.3442, λ = 0.7279, µ = 1×${e}^{-8},$ *n* = 4.682	${k}_{1}$= 9.9962$, {k}_{2}$ = 10$, {k}_{3}$ = 9.984$, {k}_{4}$ = 4.039$, {k}_{t}$ = 5.5703$, {k}_{i}$ = 10$, {k}_{d}$ = 9.987, λ = 0.3176, µ = 0.3844, *n* = 1	${k}_{1}$= 10$, {k}_{2}$ = 9.9846$, {k}_{3}$ = 7.561$, {k}_{4}$ = 1.6455$, {k}_{t}$ = 6.771$, {k}_{i}$ = 9.9041$, {k}_{d}$ = 8.2064, λ = 0.8804, µ = 1×${e}^{-8},$ *n* = 4.25	0.00188

The values of maximum overshoots (${O}_{sh}$) and maximum undershoots (${U}_{sh}$) obtained from the various considered dynamic responses of the analyzed power grid using the recommended controller relied on the COA method are considerably lower than the results obtained with other mentioned controllers via various different techniques, as shown in Fig. 12. Furthermore, Table 5 provides an in-depth overview of the system performance in Case A.1. Then, Table 6 further highlights the percentage improvements in ${O}_{sh}$ and ${U}_{sh}$ for this specific case, illustrating the enhancements achieved compared to the previous controllers.

**Table 5:** Specifications of the analyzed system's transient response for scenario A.1.

Controller structures	Considered dynamic response of (${\Delta {\text{f}}}_{{\text{a}} })$	Considered dynamic response of (${\Delta {\text{f}}}_{{\text{b}} })$	Considered dynamic response of (${\Delta {\text{P}}}_{{\text{tie}}}$)
PID-optimized via AOA^18^	${O}_{sh}$= 1.158	${O}_{sh}$= 0.0209	${O}_{sh}$= 0.01107
	${U}_{sh}$ = − 11.42	${U}_{sh}$ = − 4.443	${U}_{sh}$ = − 1.249
FOPID-optimized via ESAOA^18^	${O}_{sh}$= 0.0802	${O}_{sh}$= 0.0018	${O}_{sh}$= 0.00109
	${U}_{sh}$ = − 8.344	${U}_{sh}$ = − 2.456	${U}_{sh}$ = − 0.7403
PD-PI-optimized via HHO^3^	${O}_{sh}$= 0.7113	${O}_{sh}$= 0.02308	${O}_{sh}$= 0.0137
	${U}_{sh}$ = − 5.06	${U}_{sh}$ = − 0.760	${U}_{sh}$ = − 0.2735
FO-(PD-PI)-optimized via MRFO^3^	${O}_{sh}$= 0.0249	${O}_{sh}$= 0	${O}_{sh}$= 0
	${U}_{sh}$ = − 3.734	${U}_{sh}$ = − 0.4837	${U}_{sh}$ = − 0.1623
Fuzzy-PID + (T ${I}^{\lambda }{D}^{\mu }$)-optimized via COA	${O}_{sh}$= 0.1983	${O}_{sh}$= 0.0203	${O}_{sh}$= 0.0083
	${U}_{sh}$ = − 0.4975	${U}_{sh}$ = − 0.1839	${U}_{sh}$ = − 0.08528

All values are multiplied by (${10}^{-3}$).

**Table 6:** Percentage improvements in ${O}_{sh}$ and ${U}_{sh}$ for Case A.1.

Controller structures	${\Delta {\text{f}}}_{{\text{a}}}$		${\Delta {\text{f}}}_{{\text{b}}}$		${\Delta {\text{P}}}_{{\text{tie}}}$	
	${U}_{sh}$	${O}_{sh}$	${U}_{sh}$	${O}_{sh}$	${U}_{sh}$	${O}_{sh}$
FOPID-optimized via ESAOA^18^	26.93	93.07	44.72	91.39	40.73	90.15
PD-PI-optimized via HHO^3^	55.69	38.58	82.89	10.43	78.1	23.76
FO-(PD-PI)-optimized via MRFO^3^	67.3	**97.84**	89.11	**100**	87	**100**
Fuzzy-PID + (T ${{\varvec{I}}}^{{\varvec{\lambda}}}{{\varvec{D}}}^{{\varvec{\mu}}}$)-optimized via COA	**95.64**	82.88	**95.86**	2.87	**93.17**	25.02

Optimal percentage values are highlighted in bold format.

**Figure 13:**
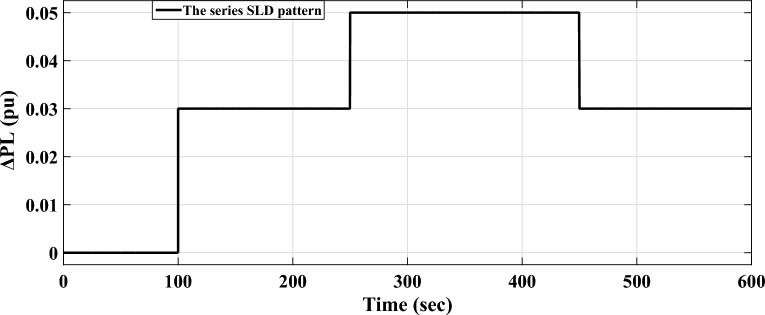
The implemented area-a series SLD form.

**Figure 14:**
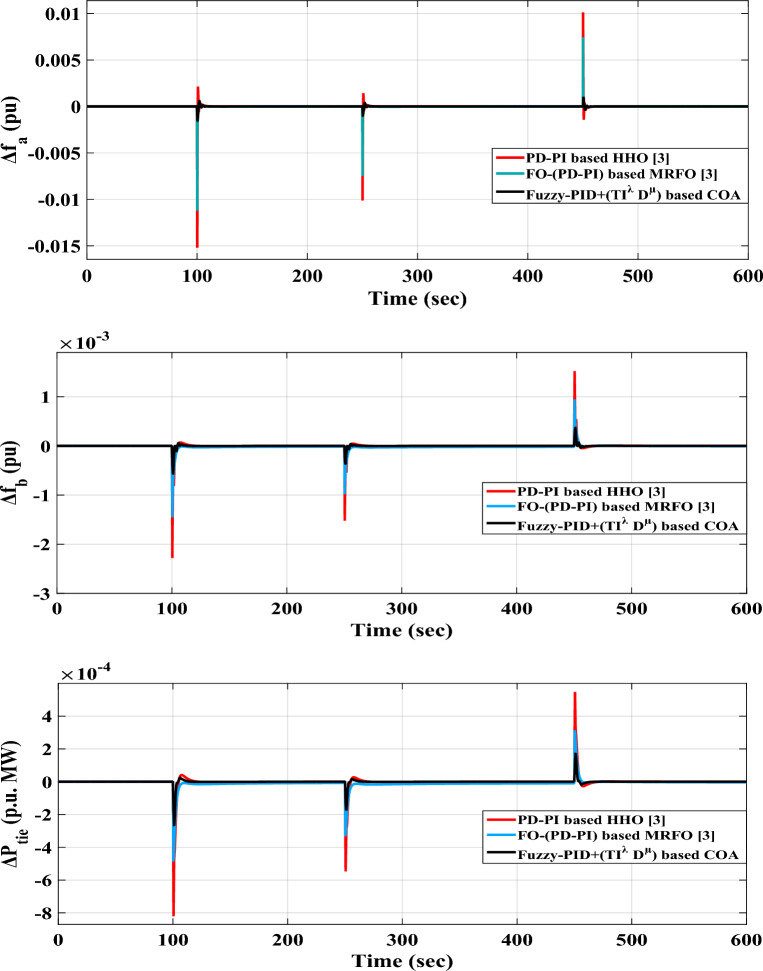
LFC controllers' dynamic responses to series step load disruption for Case A.2.

*Case A.2* A series SLD pattern is applied in this case to assess the performance of the proposed (fuzzy-PID) + (T ${I}^{\lambda }{D}^{\mu }$) controller. This pattern involves a succession of forced switches of generators or outages of connected loads, which can potentially lead to power grid instability. Figure 13 depicts the series SLD form. Then, Fig. 14 presents the system's various dynamic responses for this case.

Figure 14 illustrates that the maximum ${O}_{sh}$ and maximum ${U}_{sh}$ values obtained from the system's dynamic responses using the proposed (fuzzy-PID) + (T ${I}^{\lambda }{D}^{\mu }$) controller relied on COA are significantly lower than the results obtained from the aforementioned published controllers. Furthermore, Table 7 provides the precise values of ${O}_{sh}$ and ${U}_{sh}$ for the power flowing in the tie-line and frequencies for both areas. Additionally, Table 8 highlights the system's overall improvement percent achieved in this case.

**Table 7:** Specifications of the analyzed system's transient response for scenario A.2.

Controller structures	Considered dynamic response of (${\Delta {\text{f}}}_{{\text{a}} })$	Considered dynamic response of (${\Delta {\text{f}}}_{{\text{b}} })$	Considered dynamic response of (${\Delta {\text{P}}}_{{\text{tie}}}$)
PD-PI-optimized via HHO ^3^	${O}_{sh}$= 10.121	${O}_{sh}$= 1.52	${O}_{sh}$= 0.5469
	${U}_{sh}$ = − 15.181	${U}_{sh}$ = − 2.28	${U}_{sh}$ = − 0.82035
FO-(PD-PI)-optimized via MRFO^3^	${O}_{sh}$= 7.45	${O}_{sh}$= 0.94914	${O}_{sh}$= 0.31477
	${U}_{sh}$ = − 11.203	${U}_{sh}$ = − 1.451	${U}_{sh}$ = − 0.48686
Fuzzy-PID + (T ${I}^{\lambda }{D}^{\mu }$)-optimized via COA	${O}_{sh}$= 1.12	${O}_{sh}$= 0.3051	${O}_{sh}$= 0.1670
	${U}_{sh}$ = − 1.817	${U}_{sh}$ = − 0.5570	${U}_{sh}$ = − 0.2364

All values are multiplied by (${10}^{-3}$).

**Table 8:** Percentage improvements in ${O}_{sh}$ and ${U}_{sh}$ for Case A.2.

Controller structures	${\Delta {\text{f}}}_{{\text{a}}}$		${\Delta {\text{f}}}_{{\text{b}}}$		${\Delta {\text{P}}}_{{\text{tie}}}$	
	${U}_{sh}$	${O}_{sh}$	${U}_{sh}$	${O}_{sh}$	${U}_{sh}$	${O}_{sh}$
FO-(PD-PI)-optimized via MRFO^3^	26.2	26.39	36.36	37.56	40.65	42.44
Fuzzy-PID + (T ${{\varvec{I}}}^{{\varvec{\lambda}}}{{\varvec{D}}}^{{\varvec{\mu}}}$)-optimized via COA	**88.03**	**88.93**	**75.57**	**79.93**	**71.18**	**69.46**

Optimal percentage values are highlighted in bold format.

**Figure 15:**
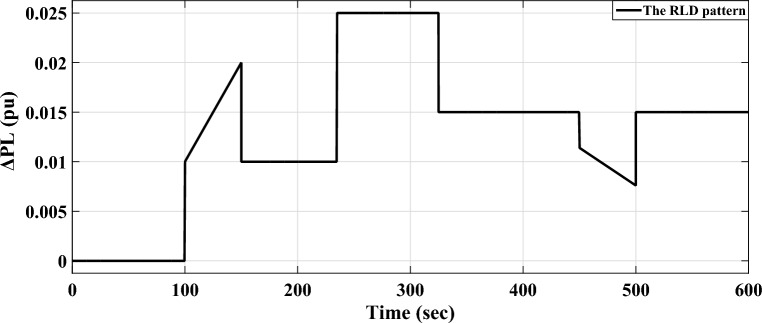
The implemented area-a RLD form.

**Figure 16:**
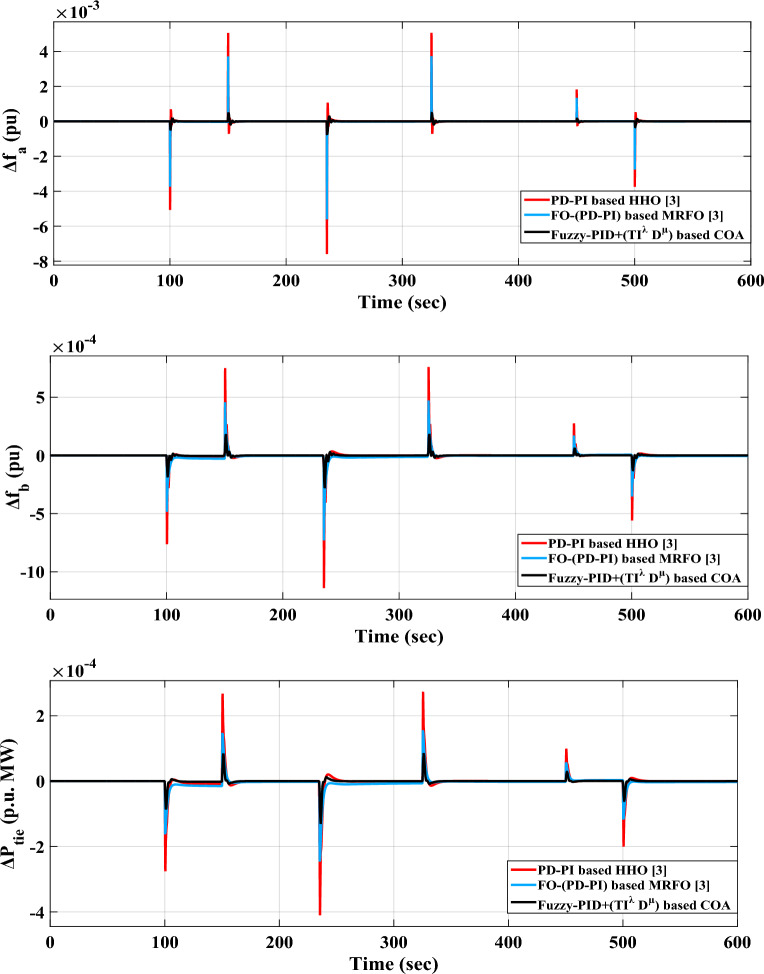
LFC controllers' dynamic responses to RLD for Case A.3.

*Case A.3* Additionally, in area-a of the analyzed power grid, a random load fluctuation form is assumed, occurring at t = 100 s. This form represents a wide range of disruptions in series in industrially interconnected loads, producing similar grid effects, such as grid instability and the occurrence of blackouts. Figure 15 clarifies the applicable RLD pattern. Furthermore, Fig. 16 provides a comparative analysis of the examined power grid’s dynamic performance according to the recommended controller scheme and the other two aforementioned controllers.

Figure 16 shows that the maximum ${O}_{sh}$ and maximum ${U}_{sh}$ values obtained from the considered power grid’s dynamic responses utilizing the recommended (fuzzy-PID) + (T ${I}^{\lambda }{D}^{\mu }$) controller relied on COA are significantly lower than the values obtained from the aforementioned published controllers. Furthermore, Table 9 shows the precise values of ${O}_{sh}$ and ${U}_{sh}$ for the power flowing in the tie-line and frequencies for both areas. Table 10 also shows the system's overall improvement percent.

**Table 9:** Specifications of the analyzed system's transient response for scenario A.3.

Controller structures	Considered dynamic response of (${\Delta \mathbf{f}}_{\mathbf{a}})$	Considered dynamic response of (${\Delta \mathbf{f}}_{\mathbf{b}\boldsymbol{ }})$	Considered dynamic response of (${\Delta \mathbf{P}}_{\mathbf{t}\mathbf{i}\mathbf{e}}$)
PD-PI-optimized via HHO^3^	${O}_{sh}$= 5.06	${O}_{sh}$= 0.7597	${O}_{sh}$= 0.27321
	${U}_{sh}$ = − 7.591	${U}_{sh}$ = − 1.14	${U}_{sh}$ = − 0.41013
FO-(PD-PI)-optimized via MRFO^3^	${O}_{sh}$= 3.722	${O}_{sh}$= 0.4714	${O}_{sh}$= 0.1557
	${U}_{sh}$ = − 5.606	${U}_{sh}$ = − 0.72973	${U}_{sh}$ = − 0.24566
Fuzzy-PID + (T ${I}^{\lambda }{D}^{\mu }$)-optimized via COA	${O}_{sh}$= 0.664	${O}_{sh}$= 0.20182	${O}_{sh}$= 0.08012
	${U}_{sh}$ = − 0.795	${U}_{sh}$ = − 0.3076	${U}_{sh}$ = − 0.1574

All values are multiplied by (${10}^{-3}$).

**Table 10:** Percentage improvements in ${O}_{sh}$ and ${U}_{sh}$ for Case A.3.

Controller structures	${\Delta {\text{f}}}_{{\text{a}}}$		${\Delta {\text{f}}}_{{\text{b}}}$		${\Delta {\text{P}}}_{{\text{tie}}}$	
	${U}_{sh}$	${O}_{sh}$	${U}_{sh}$	${O}_{sh}$	${U}_{sh}$	${O}_{sh}$
FO-(PD-PI)-optimized via MRFO^3^	26.15	26.44	35.99	37.95	40.10	43.01
Fuzzy-PID + (T ${I}^{\lambda }{D}^{\mu }$)-optimized via COA	**89.53**	**86.88**	**73.02**	**73.43**	**61.62**	**70.67**

Optimal percentage values are highlighted in bold format.

*Scenario B* Evaluating the examined power grid’s performance involving high renewables penetration, aiming to examine the grid's capability to handle this issue.

**Figure 17:**
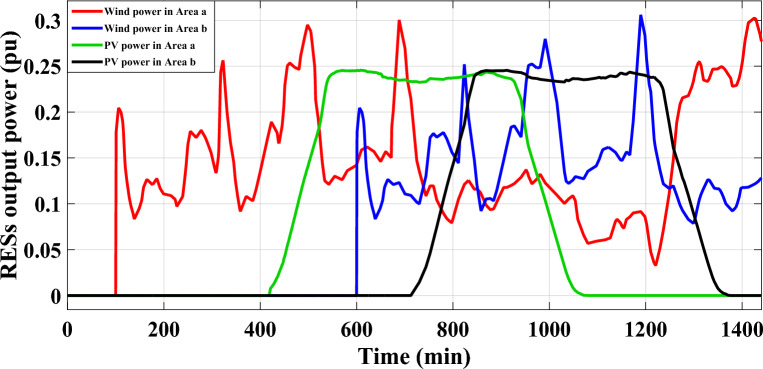
The high renewable energy penetration's generated output power in areas (**a**) and (**b**) for Case B.

**Figure 18:**
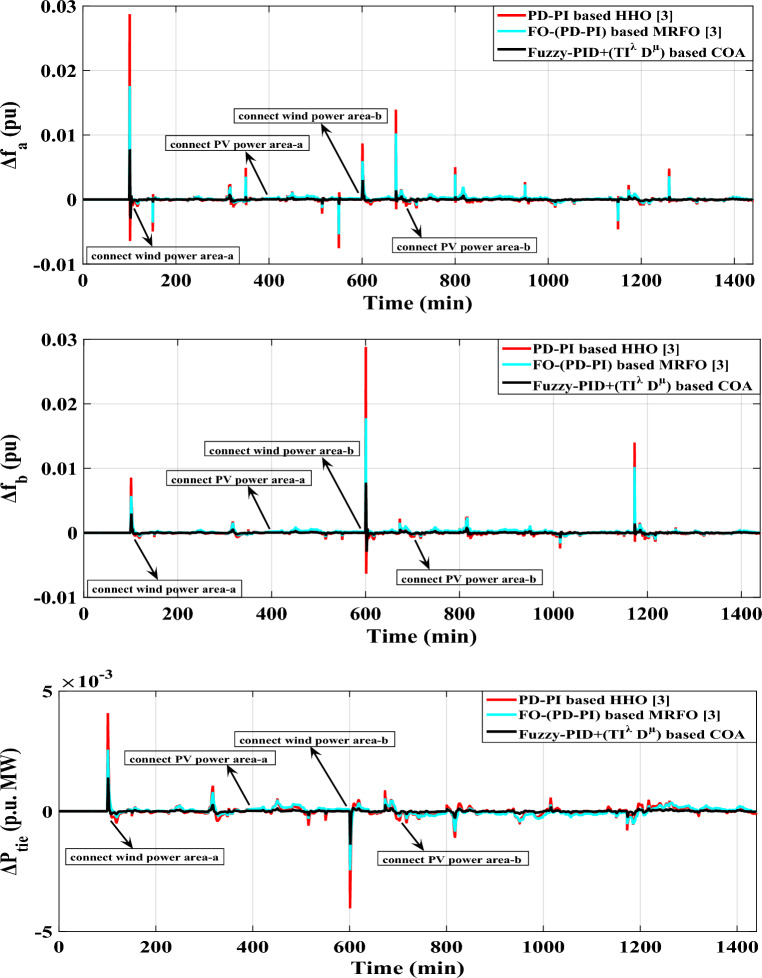
LFC controllers' dynamic responses to high penetration levels of RESs and RLD pattern for Case B.

The scenario’s purpose is to examine the disturbances caused by a significant penetration of renewables in both of the analyzed power grid's areas. Specifically, area-a has been incorporated by 10 windmills and 10 PV power plants at 100 min and 400 min, respectively, utilizing a random demand fluctuation pattern. In area-b, the identical integration parameters for the penetration of renewables are applied; PV power plants integrate at 700 min and windmills at 600 min. The generated power from the RESs integrated into the analyzed power grid, as well as various system dynamic responses, are elucidated in Figs. 17,18, respectively.

Furthermore, Table 11 shows the different acquired values of ${O}_{sh}$ and ${U}_{sh}$ for the power flowing in the tie-line and frequencies for both areas applying all of the previously mentioned controllers. Table 12 also clarifies the system's overall improvement percent for this case.

**Table 11:** Specifications of the analyzed system's transient response for scenario B.

Controller structures	Considered dynamic response of (${\Delta \mathbf{f}}_{\mathbf{a}\boldsymbol{ }})$	Considered dynamic response of (${\Delta \mathbf{f}}_{\mathbf{b}\boldsymbol{ }})$	Considered dynamic response of (${\Delta \mathbf{P}}_{\mathbf{t}\mathbf{i}\mathbf{e}}$)
PD-PI-optimized via HHO^3^	${O}_{sh}$= 28.742	${O}_{sh}$= 28.814	${O}_{sh}$= 4.082
	${U}_{sh}$ = − 7.54	${U}_{sh}$ = − 6.32	${U}_{sh}$ = − 4.036
FO-(PD-PI)-optimized via MRFO^3^	${O}_{sh}$= 17.572	${O}_{sh}$= 17.774	${O}_{sh}$= 2.559
	${U}_{sh}$ = − 5.395	${U}_{sh}$ = − 1.539	${U}_{sh}$ = − 2.45
Fuzzy-PID + (T ${I}^{\lambda }{D}^{\mu }$)-optimized via COA	${O}_{sh}$= 8.115	${O}_{sh}$= 8.082	${O}_{sh}$= 1.129
	${U}_{sh}$ = − 2.334	${U}_{sh}$ = − 0.7005	${U}_{sh}$ = − 1.167

All values are multiplied by (${10}^{-3}$).

**Table 12:** Percentage improvements in ${O}_{sh}$ and ${U}_{sh}$ for Case B.

Controller structures	${\Delta {\text{f}}}_{{\text{a}}}$		${\Delta {\text{f}}}_{{\text{b}}}$		${\Delta {\text{P}}}_{{\text{tie}}}$	
	${U}_{sh}$	${O}_{sh}$	${U}_{sh}$	${O}_{sh}$	${U}_{sh}$	${O}_{sh}$
FO-(PD-PI)-optimized via MRFO^3^	28.45	38.86	75.65	38.31	39.3	37.31
Fuzzy-PID + (T ${I}^{\lambda }{D}^{\mu }$)-optimized via COA	**69.05**	**71.77**	**88.92**	**71.95**	**71.09**	**72.34**

Optimal percentage values are highlighted in bold format.

Scenario C: Evaluating the suggested controller’s robustness in real-time for stability and reliability using the IEEE-39 bus system. The validation includes considering the impact of SLD.

**Figure 19:**
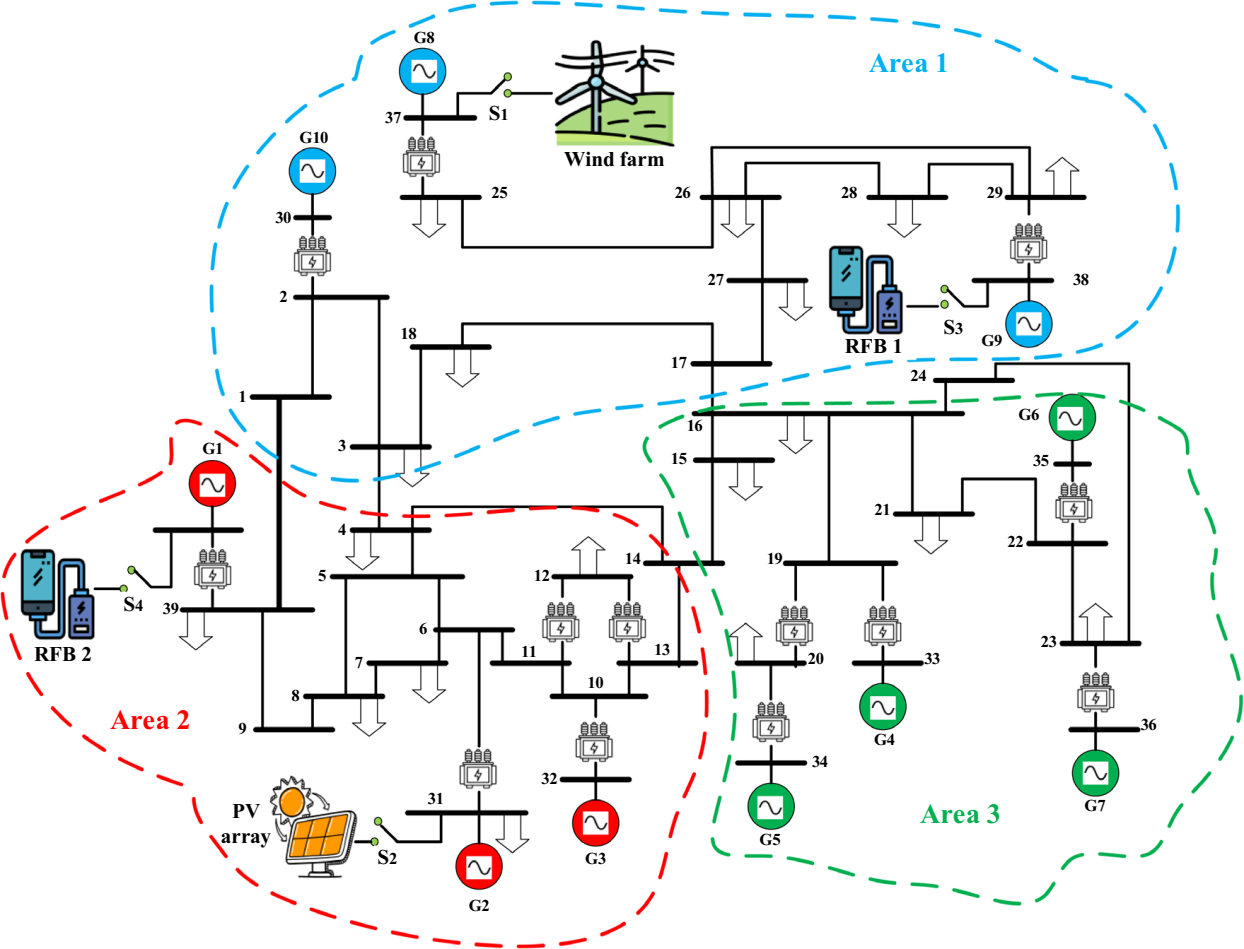
The standard IEEE-39 bus system schematic diagram.

For evaluating the suggested (fuzzy-PID) + (T ${I}^{\lambda }{D}^{\mu }$) controller approach for load frequency management, the standard real-world New England IEEE-39 bus system is applied in this scenario. The standard real-world New England IEEE-39 bus system consists of ten reheat thermal generators, nineteen loads, thirty-eight transmission lines, and twelve transformers, as shown in Fig. 19. The system is divided into three interconnected areas, namely area 1, area 2, and area 3, with respective rated area power capacities of ${P}_{r1}=1500 MW,{ P}_{r2}=2000 MW,$ and ${P}_{r3}=1500 MW$. Appendix B provides the system parameter values. In this approach, each area's generating units are substituted with a single equivalent generation unit. The following equations are used to calculate the equivalent inertia constant and speed regulation parameters^54^:

37 $${H}_{eqv}=\frac{{H}_{1}{S}_{1}+{ H}_{2}{S}_{2}+\dots +{H}_{n}{S}_{n}}{{S}_{system}}$$

38 $${R}_{evq}=\frac{1}{\frac{1}{{R}_{1}}\left(\frac{{S}_{1}}{{S}_{system}}\right)+\frac{1}{{R}_{2}}\left(\frac{{S}_{2}}{{S}_{system}}\right)+\dots +\frac{1}{{R}_{n}}\left(\frac{{S}_{n}}{{S}_{system}}\right)}HZ/{MW}_{pu}$$

39 $${S}_{system}={S}_{1}+{S}_{2}+\dots +{S}_{n}$$

where ${S}_{i},$ ${R}_{i}$ and ${H}_{i}$ refer the specified generation unit’s power rating, the factors of both speed regulation and inertia constant respectively related to ${i}^{th}$ generation units.

**Figure 20:**
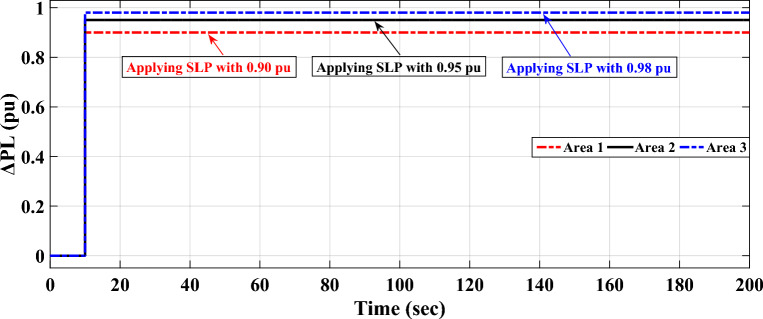
Different load disturbances applied in the three areas.

**Figure 21:**
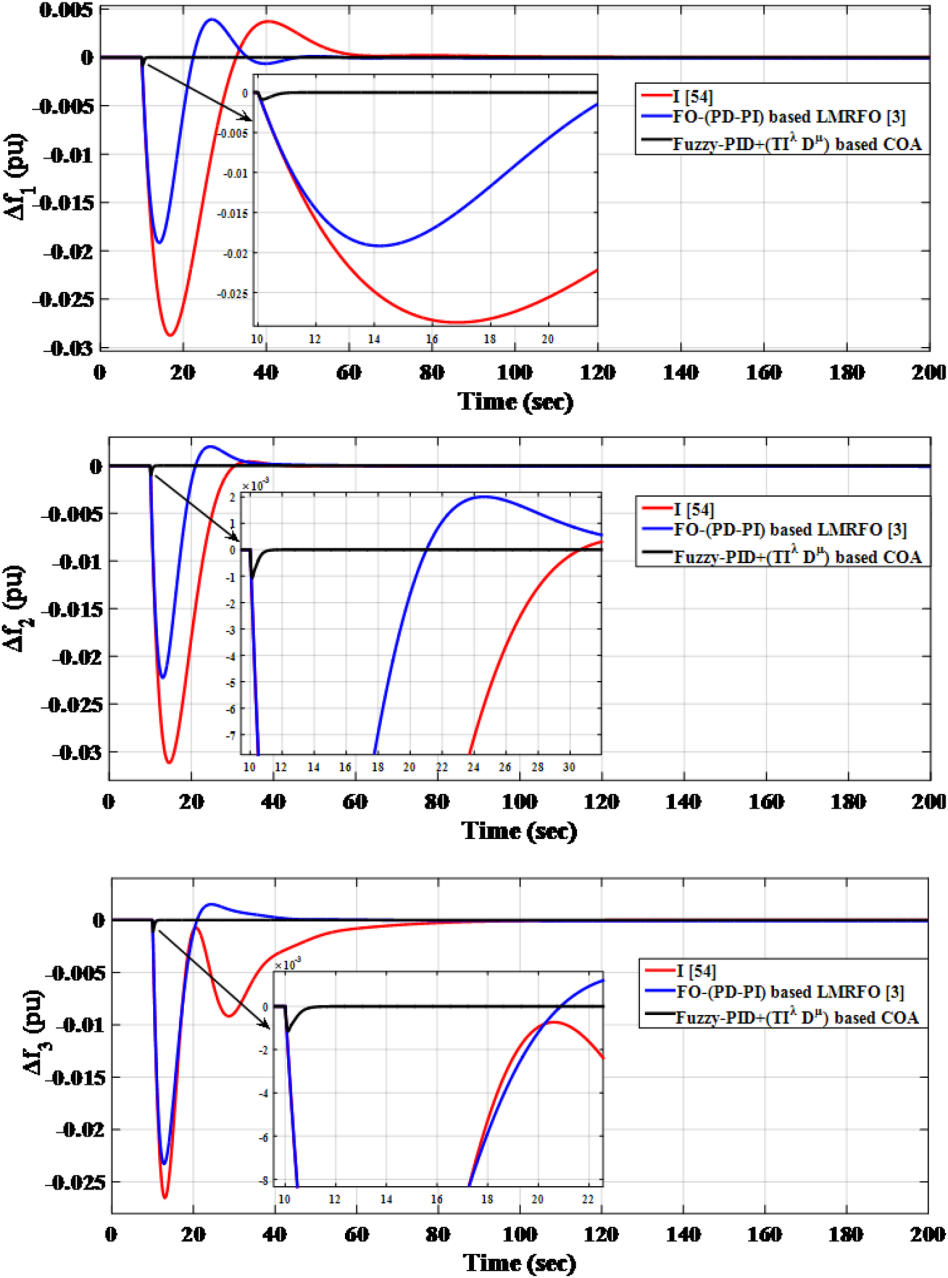

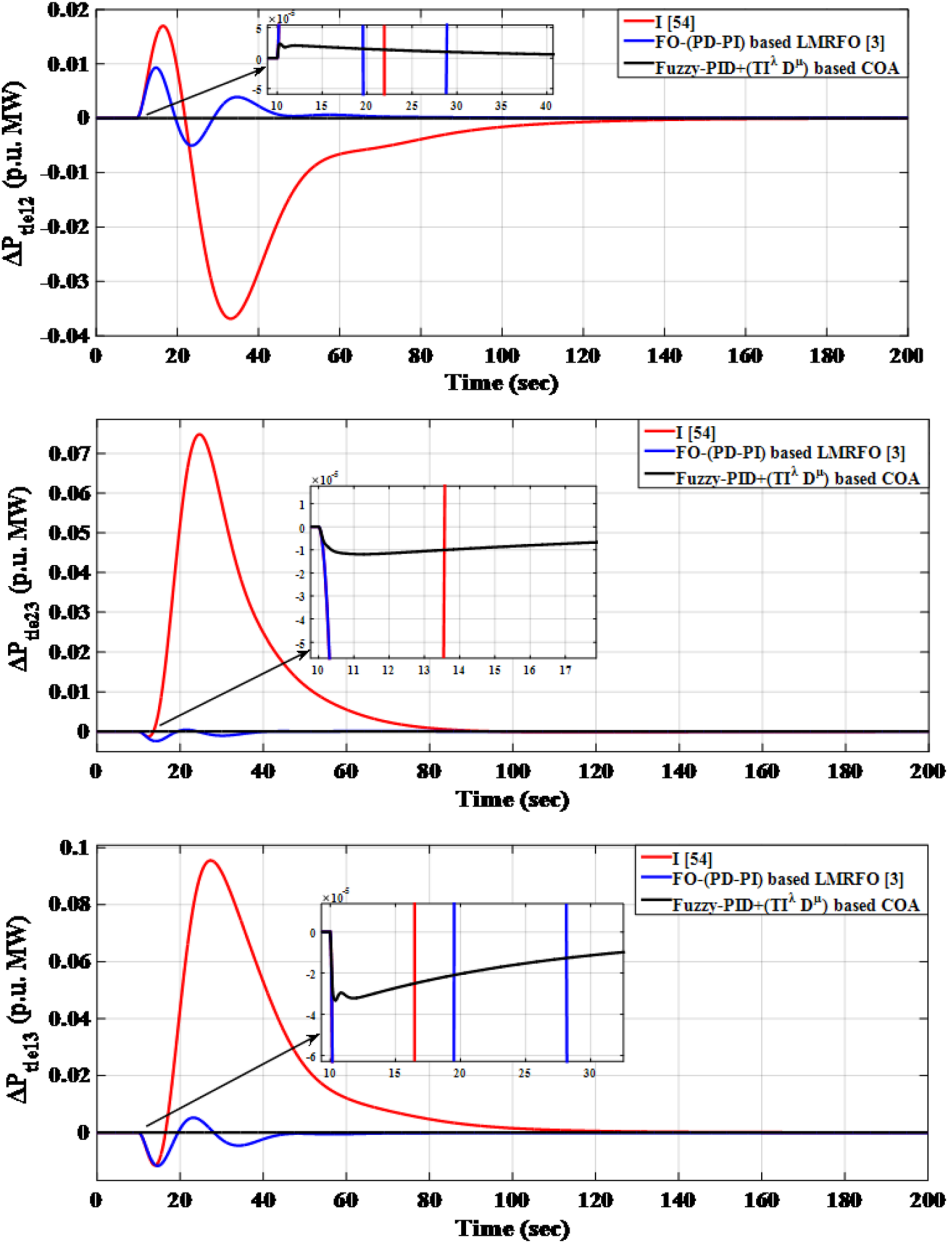
LFC controllers' dynamic responses considering SLD in a standardized IEEE system for Case C.

The simulation results, using the optimized parameters of the suggested controller listed in Table 13, show improved performance compared to previous studies. The frequencies of the three interconnected areas and the power exchange in various tie lines demonstrate shorter settling times, decreased overshoots, and undershoot. Specifically, the suggested controller outperforms the I controller and FO-(PD-PI) controller used in^3,54^ respectively. Figure 20 displays the distinct SLD profiles experienced by the three interconnected areas. The dynamic responses of the system, presented in Fig. 21, highlight the proposed controller's substantial performance in minimizing excursions and preserving system stability.

**Table 13:** The optimal optimized coefficients of the recommended controller for scenario C**.**

Controller structures	Local area 1	Local area 2	Local area 3
Fuzzy-PID + (T ${I}^{\lambda }{D}^{\mu }$)-optimized via COA	${k}_{1}$= 6.6413$, {k}_{2}$ = 8.996$, {k}_{3}$ = 6.1475$, {k}_{4}$ = 5.4056$, {k}_{t}$ = 4.8275$, {k}_{i}$ = 5.3264$, {k}_{d}$ = 5.98, λ = 0.8059, µ = 0.4159, *n* = 8.0807	${k}_{1}$= 10$, {k}_{2}$ = 1.1999$, {k}_{3}$ = 3.7147$, {k}_{4}$ = 10$, {k}_{t}$ = 9.6025$, {k}_{i}$ = 0.8318$, {k}_{d}$ = 3.6435, λ = 0.9964, µ = 0.5549, *n* = 1.2751	${k}_{1}$= 3.8123$, {k}_{2}$ = 6.779$, {k}_{3}$ = 9.8212$, {k}_{4}$ = 5.2815$, {k}_{t}$ = 9.7263$, {k}_{i}$ = 2.434841$, {k}_{d}$ = 7.913, λ = 0.5881, µ = 0.1521, *n* = 6.9069

Figure 21 depicts that the proposed controller improves the system performance by 96.43% and 94.74% when compared to the I controller and FO-(PD-PI) controller, respectively.

Scenario D: Evaluating the efficacy of the recommended controller utilizing a real-time IEEE-39 bus system, considering high renewables penetration within the system, to assess its effectiveness with the goal to attain dependability and stability.

**Figure 22:**
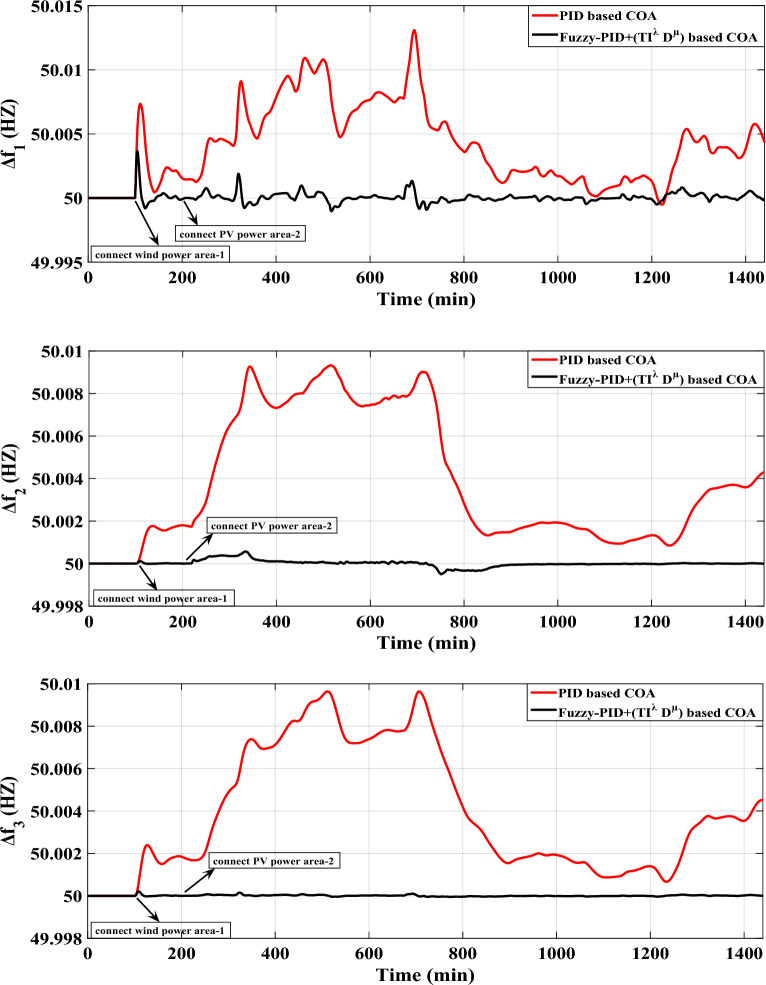
LFC controllers' considered dynamic responses with high renewables penetration in a standardized IEEE system for Case D.

Similarly, this scenario involves the suggested controller’s real-time validation using the IEEE-39 bus system, but with a specific focus on diminishing frequency excursions. The evaluation takes into account the high renewable energy penetrations within the system as depicted in Fig. 19. In this study, ten wind power plants and ten PV power plants have penetrated into areas 1 and 2 of the IEEE-39 bus system via switches (s1) and (s2) respectively. Figure 22 depicts a fair maiden comparison clarifying the superior performance of the proposed controller in attaining the system frequency to a closely predefined value (≈50 HZ) over the performance of PID controller tuned by COA. Table 14 presents the obtained optimal PID controller parameters optimized by COA.

**Table 14:** The Optimum optimized PID controller coefficients for scenario D.

Controller structures	Local area 1	Local area 2	Local area 3
PID-optimized via COA	${k}_{p}$= 9.9789$, {k}_{i}$ = 8.6957$, {k}_{d}$ = 6.8607	${k}_{p}$= 8.8642$, {k}_{i}$ = 2.2517$, {k}_{d}$ = 3.9077	${k}_{p}$= 2.2822$, {k}_{i}$ = 0.3942, ${k}_{d}$ = 2.0487

Figure 22 shows that the proposed controller strengthens the system performance by 75.19% when compared to the PID controller tuned via COA considering high renewables penetration in the studied system.

Scenario E: Evaluating the performance of the proposed controller under varying CDT conditions before and after the LFC in the IEEE-39 bus system. This evaluation also considers high renewable energy penetrations.

**Figure 23:**
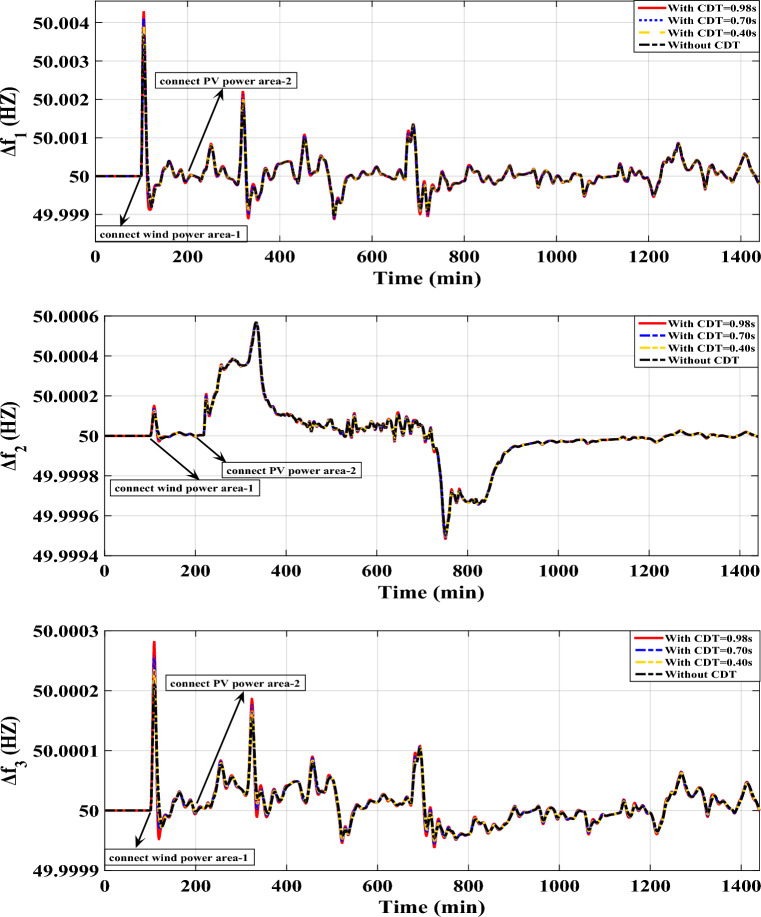
LFC controllers' considered dynamic responses with high renewables penetration and CDT challenge in a standardized IEEE system for Case E.

To validate the proposed controller, experiments were conducted on the IEEE-39 bus system. The system was designed to incorporate high penetration levels RESs. In these experiments, different CDT values were introduced before and after the supplementary controllers. When the CDT challenge is executed, Fig. 23 provides a sufficient comparison of the considered system dynamic responses.

The proposed controller demonstrates its capability to effectively handle the perturbations caused by RESs and the challenges posed by CDT. It successfully maintains the frequencies of different areas within predefined values. This highlights the proposed controller’s durability in addressing the complexities introduced by RESs and communication delays, ultimately ensuring stable and reliable operation of the system.

Scenario F: Evaluating the proposed strategy’s effectiveness by comparing the effectiveness of the IEEE 39 buses system with and without the proposed strategy, considering high renewables penetration.

**Figure 24:**
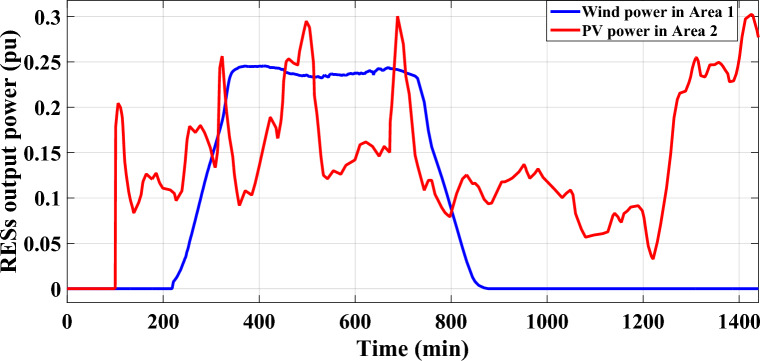
The controllable generated power of the CRFBs systems in both local areas for Case F.

**Figure 25:**
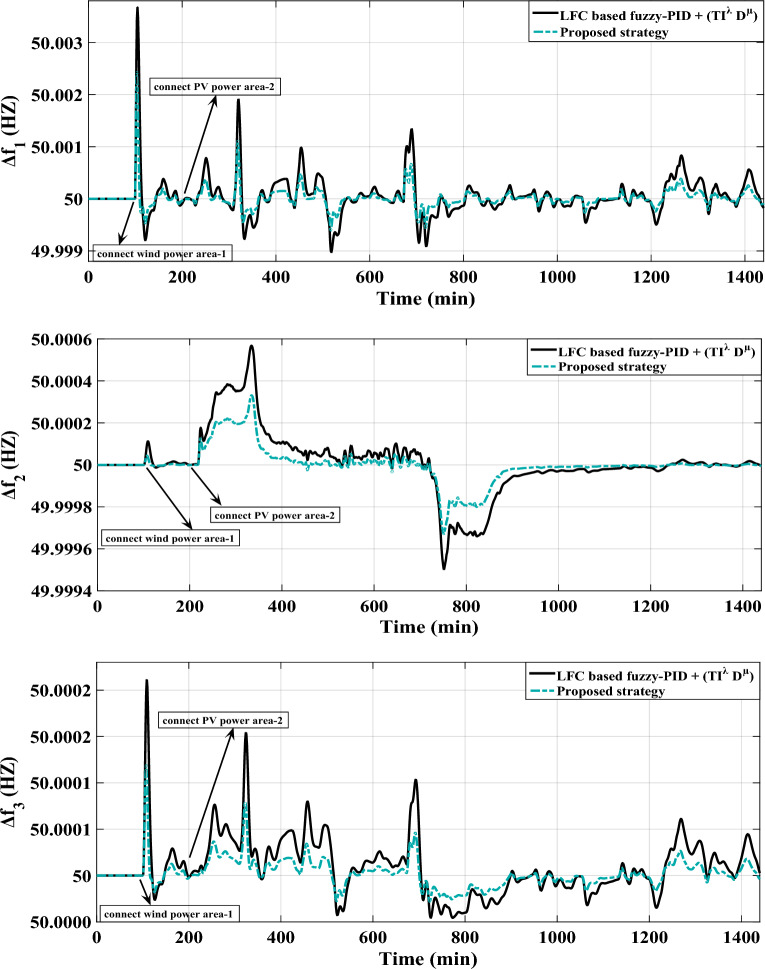
LFC controllers' considered dynamic responses considering a coordinated strategy besides high renewables penetration in the IEEE-39 bus system for Case F.

In this specific scenario, the IEEE-39 bus system performance is examined while considering a significant integration of RESs. Whereas, wind energy is integrated into area 1 at t = 100 min, and PV energy is integrated into area 2 at t = 200 min. The proposed coordinated strategy involves the incorporation of CRFBs systems in area 1 and area 2. These CRFBs systems work collaboratively by closing switches (s3) and (s4) over different simulation durations. When wind energy penetrates area 1, the CRFBs included in area 1 share their active power with the assessed power system. Additionally, when PV energy penetrates area 2, the CRFBs included in area 2, share their extra active power with the assessed power system. The CRFBs utilize stored energy to enhance LFC and optimize the overall performance of the power system under unusual circumstances. The major objective is to regulate the system frequency, which is prone to considerable fluctuations as a result of the high renewables penetration in both areas. Table 15 illustrates the PID controller variables that were obtained for the proposed strategy. Figure 24 depicts the CRFBs systems'-controlled output signal, which denotes the outcome of the active power quantity. Additionally, Fig. 25 compares the total dynamic responses of the entire system in the IEEE-39 bus system with and without the proposed strategy.

**Table 15:** The optimum values of the PID controller tuned by COA for the proposed strategy.

Controller Structures	Local area 1	Local area 2
PID-optimized via COA	${k}_{p}$= 10$, {k}_{i}$ = 10$, {k}_{d}$ = 0.0001	${k}_{p}$= 8.4772$, {k}_{i}$ = 4.4128$, {k}_{d}$ = 5.4788

Figure 25 indicates that the proposed strategy, which includes the LFC besides CRFBs, demonstrates its capability to successfully handle the perturbations caused by RESs in the studied system. It successfully maintains the frequencies of different areas within predefined values. Whereas, the proposed strategy improves the system performance by 50% as compared to without it.

## *Scenario G* Stability analysis of the proposed (fuzzy-PID) + (T ${I}^{\lambda }{D}^{\mu }$) controller

**Figure 26:**
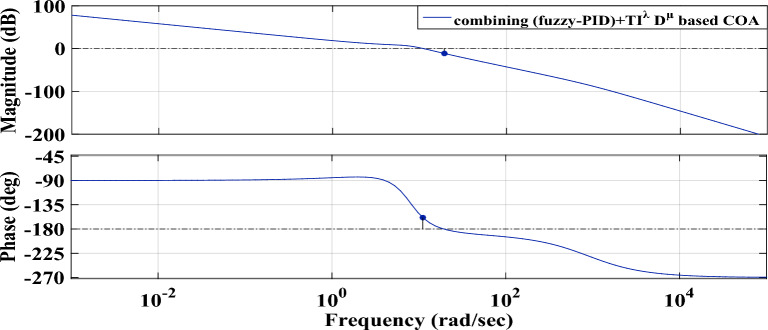
The bode plot of the power grid loop gains with the proposed (fuzzy-PID) + (T ${I}^{\lambda }{D}^{\mu }$) controller.

Figure 26 illustrates the bode plot of the loop gains of the analyzed power grid using the proposed (fuzzy-PID) + (T ${I}^{\lambda }{D}^{\mu }$) controller. The magnitude of the gain margin plot is observed to be more stable across all frequencies when employing the proposed controller. Additionally, the phase margin shows an infinite value, indicating that the suggested controller is capable of effectively handling system uncertainties.

## Conclusion

This study has presented significant findings that contribute to power system stability when transitioning from traditional power stations to renewable energy sources (RESs). Firstly, an effective structure has been presented to ensure stable frequency in the power system during this transition. This structure combines the improved load frequency controller (LFC) and controlled redox flow batteries (CRFBs) to effectively manage frequency fluctuations in considered grid. Furthermore, the controller in LFC depend on (fuzzy-PID) + (T ${I}^{\lambda }{D}^{\mu }$) controller. The proposed controller improves the system performance than more on controller such as (PID, FO-PID, PD-PI, and FO-(PD-PI)) controllers. Furthermore, the proposed controller contributes with CRFBs to maintain the system stability during high renewables. The constraints for this proposed scheme have been determined using the crayfish optimization algorithm. LFC controller effectiveness has been validated by comparing its performance to that of other published controllers. Importantly, the proposed strategy demonstrates the ability to maintain system reliability even during periods of high renewable energy penetration. The authors intend to incorporate more ESSs into the system in future research, ensuring its seamless operation throughout the day. Furthermore, as all generation sources will be renewable, the design process took into account the virtual synchronous generator.

## Supplementary Information

Supplementary Tables.

## Data Availability

All data generated or analysed during this study are included in this published article and its supplementary information file.

## Abbreviations

RESs: Renewable energy resources
PV: Photovoltaic
ESSs: Energy storage systems
SMES: Superconducting magnetic energy storage
SSSC: Static synchronous series compensator
PEVs: The plug-in electric vehicles
CRFBs: The controlled redox flow batteries
P.u: The value of the per unit
*ACE*: The area control error
$${U}_{sh}$$: Undershoot
RLD: Random load disruption
$${O}_{sh}$$: Overshoot
SLD: Step load disruption
AGC: The automatic generation control
*MW*: Mega-watt
LFC: Load frequency control
CDT: Communication delay time
FLC: Fuzzy logic control
$Tsim$: Total simulation time
$dt$: Error signal sampling interval
TID: Tilted-integral-derivative controller
*ISE*: The integral square error
PID: Proportional-integral-derivative controller
*ITAE*: The integral time absolute error
FOCs: Fractional-order-controllers
FO-(PD-PI): Fractional-order-proportional-derivative-proportional-integral controller
PD-PI: Proportional-derivative-proportional-integral controller
${{\text{TI}}}^{\uplambda }{{\text{D}}}^{\upmu }$: Tilt-fractional-order-integral-fractional-order-derivative controller
FOPID: Fractional-order PID controller
HHO: Harris hawks optimization method
MRFO: Manta ray foraging optimization method
AOA: Arithmetic optimization algorithm
*Min*: Minutes
$J$: The objective function
*Sec*: Seconds
ESAOA: Eagle strategy arithmetic optimization algorithm
COA: Crayfish optimization algorithm
LMRFO: Leader manta ray foraging optimization
$\Delta {{\text{f}}}_{{\text{a}}}$: Area a frequency fluctuation
${\Delta {\text{P}}}_{\mathrm{tie a}-{\text{b}}}$: Power flow from area-a to area-b via the tie-line
$\Delta {{\text{f}}}_{{\text{b}}}$: Area b frequency fluctuation
${\Delta {\text{P}}}_{\mathrm{tie b}-{\text{a}}}$: Power flow from area-b to area-a via the tie-line
$${T}_{ab}$$: Synchronization coefficient
${{\text{a}}}_{{\text{ab}}}$: Capacity ratio of control area
${K}_{T}$: The thermal unit’s participation factor
${B}_{a}$: Area a’s frequency bias
${K}_{G}$: The gas turbine’s participation factor
${B}_{b}$: Area b’s frequency bias
${K}_{H}$: The hydropower plant’s participation factor
${T}_{cr}$: Time delay regarding gas turbine combustion reaction
${Y}_{c}$: Lag time constant regarding the speed governor of gas turbine
${b}_{g}$: The gas turbine constant of valve positioner
${c}_{g}$: The valve positioner of the gas turbine
${T}_{cd}$: Discharge volume-time constant regarding gas turbine compressor
${T}_{fc}$: The fuel time constant of the gas turbine
${X}_{c}$: Lead time constant regarding the speed governor of gas turbine
${T}_{r}$: The time constant of reheater steam turbine
${T}_{t}$: The time constant of turbine
${K}_{r}$: The gain of reheater steam turbine
${R}_{1}$: The value of thermal turbine regulating constant
λ: The integrator’s fractional-order
${R}_{2}$: The value of hydropower plant regulating constant
$\upmu$: The differentiator's fractional-order
${R}_{3}$: The value of gas turbine regulating constant
${T}_{sg}$: The time constant of governor
${P}_{wt}$: Generated wind turbine power
${V}_{W}$: The speed of wind
${C}_{p}$: The coefficient of rotor blades
${\omega }_{T}$: Speed of the rotor
${A}_{T}$: A turbine's swept area
*Ρ*: The density of air
${r}_{T}$: The rotor radius
$${C}_{1}$$*-*$${C}_{7}$$: Turbine coefficients
*Β*: The pitch angle
${\lambda }_{i}$: The intermittent tip-speed ratio
${\lambda }_{T}$: The optimum tip-speed ratio
${T}_{rs}$: The speed governor reset time of the hydro turbine
${T}_{rh}$: The transient droop time constant of the hydro turbine speed governor
${T}_{ps}$: The time constant of power system
${K}_{ps}$: The gain constant of power system
${T}_{gh}$: The speed governor time constant of hydro turbine
${T}_{w}$: The nominal string time of water in penstock
