# Supplementary material for: Frequency regulation in a hybrid renewable power grid: an effective strategy utilizing load frequency control and redox flow batteries
Source: Sci Rep. 2024 Apr 26;14:9576. doi: 10.1038/s41598-024-58189-2 (PMC11522519; doi:10.1038/s41598-024-58189-2)
Supplement: Supplementary file 1 — Supplementary Tables. [file 41598_2024_58189_MOESM1_ESM.docx]

**Appendix A**

**Table A.1:** The included parameter values in the analyzed power grid.

| **Symbol** | **Standard Value** |
| --- | --- |
| $B_{i}$ | 0.4312 MW/Hz |
| $T_{ab}$ | 0.0433 MW |
| $R_{1}$  $R_{2}$  $R_{3}$ | 2.4 HZ/MW  2.4 HZ/MW  2.4 HZ/MW |
| $a_{\mathrm{ab}}$ | −1 |
| $K_{T}$ | 0.543478 |
| $K_{H}$ | 0.326084 |
| $K_{G}$ | 0.130438 |
| $K_{ps}$ | 68.9566 |
| $T_{ps}$ | 11.49 s |
| $T_{sg}$ | 0.08 s |
| $T_{t}$ | 0.3 s |
| $K_{r}$ | 0.3 |
| $T_{r}$ | 10 s |
| $T_{gh}$ | 0.2 s |
| $T_{rs}$ | 5 s |
| $T_{rh}$ | 28.75 s |
| $T_{w}$ | 1 s |
| $b_{g}$ | 0.05 |
| $c_{g}$ | 1 |
| $Y_{c}$ | 1 s |
| $X_{c}$ | 0.6 s |
| $T_{cr}$ | 0.01 s |
| $T_{fc}$ | 0.23 s |
| $T_{cd}$ | 0.2 s |

**Appendix B**

**Table B.1:** The temperature of the solar cell under varying radiation conditions.

| **The radiation ((W/**$\mathbf{m}^{\mathbf{2}}$**)** | **The temperature of solar cell (**$\mathbf{℃}$**)** | |
| --- | --- | --- |
| 582.13 | 36.78 | |
| 595.18 | 37.38 | |
| 626.32 | 38.3 | |
| 668.56 | 39.22 | |
| 714.235 | 40.175 | |
| 760.48 | 41.025 | |
| 790.78 | 42.23 | |
| 815.61 | 44.615 | |
| 820.4725 | 46.395 | |
| 840.4825 | 47.12 | |
| 841.4075 | 47.655 |  |

**Table B.2:** The RFBS parameters [45]**.**

| **Symbol** | **Standard Value** |
| --- | --- |
| $k_{p,RFBi}$ | 1.8 |
| $k_{r,RFBi}$ | 1 |
| $T_{r,RFBi}$ | 0.78 |
| $T_{d,RFBi}$ | 0 |

**Table B.3:** The parameter values of IEEE 39 buses power system [53].

| **Local Area** | **Generating unit** | $\boldsymbol{H}$ | $\boldsymbol{R}$ | $\boldsymbol{D}$ | $\boldsymbol{T}_{\boldsymbol{g}}$ | $\boldsymbol{T}_{\boldsymbol{t}}$ |
| --- | --- | --- | --- | --- | --- | --- |
| 1 | $1$  2  3 | 70.0  30.0  35.8 | $0.05$  0.05  0.05 | 1  1  1 | 0.08  0.08  0.08 | 0.04  0.04  0.04 |
| 2 | 4  5  6  7 | 28.6  26  34.8  26.4 | 0.05  0.05  0.05  0.05 | 1  1  1  1 | 0.08  0.08  0.08  0.08 | 0.04  0.04  0.04  0.04 |
| 3 | 8  9  10 | 24.3  34.5  20.0 | 0.05  0.05  0.05 | 1  1  1 | 0.08  0.08  0.08 | 0.04  0.04  0.04 |
